# Engineering Chitosan-Functionalized Magnetopolymeric Nanoparticles as a Theranostic Platform for MRI-Guided Magnetic Hyperthermia

**DOI:** 10.3390/nano16161013

**Published:** 2026-08-17

**Authors:** Silvia Fuerte-Rodríguez, Lorena García-Hevia, Juan Gallo, Manuel Bañobre-López, José L. Arias

**Affiliations:** 1 Department of Pharmacy and Pharmaceutical Technology, Faculty of Pharmacy, University of Granada, 18071 Granada, Spain; sfuerte@correo.ugr.es; 2 Department of Anatomy and Cell Biology, University of Cantabria, 39005 Santander, Spain; lorena.garciahevia@unican.es; 3 Nanomedicine Group, Marqués de Valdecilla Research Institute (IDIVAL), Avda. Cardenal Herrera Oria s/n, 39011 Santander, Spain; 4 Advanced (Magnetic) Theranostic Nanostructures Lab, Nanomedicine Group, International Iberian Nanotechnology Laboratory (INL), Avda. Mestre José Veiga, 4715-330 Braga, Portugal; juan.gallo@inl.int (J.G.); manuel.banobre@inl.int (M.B.-L.); 5 Institute of Biopathology and Regenerative Medicine (IBIMER), Biomedical Research Center (CIBM), University of Granada, 18071 Granada, Spain; 6 Biosanitary Research Institute of Granada (ibs.GRANADA), Andalusian Health Service (SAS), University of Granada, 18071 Granada, Spain

**Keywords:** magnetic nanoparticles, chitosan, poly(ethyl cyanoacrylate), magnetic hyperthermia, magnetic resonance imaging, theranostic nanoplatform

## Abstract

Magnetopolymeric nanocomposites represent promising platforms for advanced nanomedicine due to their ability to combine magnetic responsiveness with polymer-mediated biological functionality. In this study, a hybrid nanocomposite system based on maghemite (Mh), poly(ethyl cyanoacrylate) (PECA), and chitosan (Cs) was developed and systematically characterized for biomedical applications. Mh nanoparticles (NPs) were incorporated as magnetic cores, while PECA acted as a biodegradable polymeric matrix, and chitosan provided surface functionalization and enhanced biocompatibility. The nanocomposites were prepared following anionic polymerization and coacervation methods and characterized in terms of structure particle size, electrokinetics and magnetic responsiveness. The results demonstrated the formation of a nanoscale (core/shell)/shell system with superparamagnetic properties. Importantly, the nanocomposites were evaluated as magnetic resonance imaging (MRI) contrast agents, exhibiting significant transverse relaxivity. In vitro cytotoxicity assays demonstrated the absence of significant toxic effects, while ex vivo hemocompatibility studies confirmed their compatibility with blood components. Furthermore, their potential as hyperthermia agents was demonstrated in vitro under the influence of an alternating magnetic field (AMF). Overall, the (core@shell)@shell nanocomposites combined superparamagnetic functionalities with favorable biological properties, supporting their potential use as multifunctional platforms for theranostic applications, including MRI contrast enhancement and magnetically induced hyperthermia.

## 1. Introduction

Cancer remains one of the most critical global health challenges, with approximately 2,041,910 new cases and nearly 618,120 cancer-related deaths reported in the United States of America in 2025 [1]. Despite significant advances in oncology, chemotherapy continues to be a cornerstone of cancer treatment; however, its clinical efficacy is often compromised by nonspecific toxicity, severe side effects, and the development of multidrug resistance. These limitations have driven intensive research efforts toward the development of targeted therapeutic strategies capable of improving treatment selectivity while minimizing systemic toxicity [1,2].

In this context, nanotechnology has emerged as a powerful approach for cancer diagnosis and therapy, offering the possibility to engineer nanoscale systems with tailored physicochemical and biological properties. Nanomaterials can be designed to act as carriers for therapeutic agents, including small-molecule drugs, proteins, nucleic acids, and immunomodulators, thereby enhancing drug stability, improving pharmacokinetics, and increasing drug accumulation at tumor sites. Moreover, the ability to manipulate matter at the nanoscale enables the emergence of functionalities not observed in bulk materials, opening new avenues for precision oncology [3].

One of the key advantages of NP-based drug delivery systems is their ability to exploit the enhanced permeability and retention (EPR) effect, which arises from the abnormal vasculature and impaired lymphatic drainage characteristic of solid tumors. The presence of fenestrated blood vessels with defective endothelial junctions, typically ranging from 100 to 780 nm, facilitates the extravasation of NPs of appropriate size into the tumor interstitium, where prolonged retention can be achieved [4]. This passive targeting mechanism has positioned nanocarriers as promising tools for improving drug delivery and therapeutic efficacy in cancer treatment.

Among the wide range of nanomaterials investigated, polymeric NPs have attracted particular interest due to their biodegradability, biocompatibility, and versatility. Poly(alkyl cyanoacrylates) (PACAs), originally developed as surgical adhesives, were among the first biodegradable polymers used for NP-based drug delivery in humans [5]. PACA NPs exhibit several advantageous features, including straightforward formulation, ease of scale-up, good in vitro stability, and rapid biodegradation [5]. Importantly, PACA-based systems have demonstrated the ability to overcome P-glycoprotein-mediated multidrug resistance, a major obstacle in cancer chemotherapy [6]. Notably, a specific PACA-based formulation, Doxorubicin-loaded poly(isohexyl cyanoacrylate) NPs (Transdrug^®^), progressed to phase III clinical evaluation in advanced hepatocellular carcinoma, demonstrating significant therapeutic improvement, including increased survival rates (88.9% vs. 54.4% for standard treatment) and enhanced median survival in earlier clinical studies, highlighting the translational potential of this polymer family [7,8].

In parallel, magnetic NPs (MNPs), particularly iron oxide-based systems, have gained substantial attention in oncology due to their unique magnetic properties and favorable biocompatibility. Iron oxide NPs, typically with diameters below 20 nm, exhibit superparamagnetic behavior and are commonly referred to as superparamagnetic iron oxide nanoparticles (SPIONs) and can generate heat when exposed to an alternating magnetic field, enabling their application in magnetic hyperthermia therapy [9,10]. MH is based on magnetic relaxation losses, primarily governed by Néel and Brownian relaxation mechanisms, as well as hysteresis losses in larger or aggregated systems, leading to efficient dissipation of magnetic energy into heat [11,12,13]. Localized heating to temperatures around 42 °C can induce tumor cell damage while largely sparing healthy tissues, making MH an attractive cancer treatment modality [14]. Additionally, mild hyperthermia has been shown to increase tumor vascular permeability, further enhancing NP accumulation via the EPR effect [4].

Beyond therapeutic applications, SPIONs are also widely employed as contrast agents in MRI, enabling noninvasive tumor visualization and treatment monitoring, owing to their high magnetic susceptibility, which induces local magnetic field inhomogeneities and enhances transverse (*T*_2_) relaxation of surrounding protons, resulting in significant signal attenuation and improved imaging contrast [15,16,17,18].

The integration of diagnostic and therapeutic functionalities within a single nanosystem has led to the development of theranostic platforms, which allow for simultaneous tumor imaging, targeted therapy, and real-time assessment of treatment efficacy. Several iron oxide-based nanomaterials are currently under clinical evaluation, underscoring their clinical relevance [14].

To maximize the biomedical performance of SPIONs, polymer-based surface modifications are essential. Polymer coatings (e.g., PACA coating) can improve colloidal stability, prevent aggregation, enhance biocompatibility, and enable functionalization with targeting or responsive moieties [19,20]. However, extending NP circulation time remains a major challenge. While polyethylene glycol (PEG) is widely used for this purpose, the emergence of anti-PEG antibodies has raised concerns regarding accelerated blood clearance and immunogenicity [4]. As an alternative, natural polysaccharides such as Cs have attracted increasing interest. Cs is a biodegradable, biocompatible, and non-toxic polymer, bearing amino groups which confer chemical versatility and pH-responsive behavior. Interestingly, Cs exhibits enhanced mucoadhesive properties, promoting prolonged interaction with biological tissues and facilitating drug uptake [21]. Furthermore, Cs has been reported to exhibit stealth-like properties, delaying opsonization and recognition by the reticuloendothelial system [22]. Its tunable solubility, surface charge, and functionalization capacity make Cs an attractive component for the design of responsive and targeted nanocarriers [23].

Based on these considerations, the rational integration of MNPs within biodegradable polymeric matrices surface-coated with polysaccharides (e.g., Cs) represents a promising strategy for the development of multifunctional cancer nanotherapies. In this study, the development of a previously unexplored hybrid nanocomposite system based on Mh, PECA, and Cs is described. The proposed nanosystem was comprehensively characterized in terms of physicochemical, magnetic, hyperthermic, and imaging properties and evaluated in vitro for antitumor activity against HeLa cells. This multifunctional platform was designed to combine magnetic responsiveness, magnetic hyperthermia, MRI contrast enhancement, and favorable blood compatibility within a biodegradable polymeric nanocomposite, providing a promising platform for the development of future theranostic cancer nanomedicines.

Despite the remarkable progress achieved in multifunctional iron oxide nanomaterials, important challenges remain before their widespread biomedical translation. Although numerous polymer-coated SPIONs have been developed as MRI contrast agents or magnetic hyperthermia mediators, the simultaneous optimization of MRI relaxivity, magnetic heating efficiency, colloidal stability and biological performance within a single nanosystem remains challenging, as these properties are strongly influenced by magnetic core size, NP architecture and surface chemistry [24,25,26]. Moreover, while several recent studies have reported promising relaxometric or hyperthermia performance, comparatively fewer have provided a comprehensive biological evaluation including blood compatibility, despite this being a fundamental prerequisite for intravenously administered nanomedicines [26,27,28]. Consequently, the development of biodegradable multifunctional nanoplatforms capable of combining competitive imaging performance, efficient magnetic hyperthermia and favorable hemocompatibility remains an important objective in cancer theranostics.

## 2. Materials and Methods

### 2.1. Materials

All reagents employed were of analytical grade and acquired from Panreac (Barcelona, Spain), except for ethyl cyanoacrylate (ECA) (Henkel Loctite, Dublin, Ireland). Dulbecco’s modified eagle’s medium (DMEM), phosphate-buffered saline (PBS), Hank’s Balanced Salt Solution (HBSS), bovine serum albumin (BSA; heat shock fraction, 98% purity level), fetal bovine serum (FBS), and Penicillin–Streptomycin solution (containing 10,000 U/mL of Penicillin and 10 mg/mL of Streptomycin) were obtained from Merck KGaA (Darmstadt, Germany). Chitosan (MW ≈ 50,000–190,000 Da, degree of desacetylation 75–85%) was also obtained from Merck. Ultrapure water used throughout the experiments was obtained via a Milli-Q^®^ Academic system (Millipore, Madrid, Spain).

### 2.2. Methods

#### 2.2.1. Formulation Methods

The Mh nuclei were obtained by oxidation of magnetite NPs previously prepared by co-precipitation [29]. Briefly, Fe_3_O_4_ NPs were prepared by the simultaneous addition of FeCl_3_ (1 M, 40 mL) and FeCl_2_ (2 M in 2 M HCl, 10 mL) into 500 mL of 0.7 M NH_3_ under mechanical stirring (700 rpm) at room temperature for 30 min. The resulting particles were magnetically separated (400 mT), redispersed in 250 mL of 2 M HClO_4_, and stabilized for 12 h. Subsequently, Fe_3_O_4_ NPs were oxidized to Mh by dispersion in 600 mL of 0.34 M Fe(NO_3_)_3_ at 90 °C for 30 min. The final NPs were purified by repeated centrifugation (30 min, 9000 rpm) and redispersed in water until the supernatant conductivity reached ≤ 10 μS·cm^−1^.

Mh@PECA NPs were then obtained by anionic polymerization, directly onto the Mh cores dispersed in the aqueous phase [6,30]. Polymerization was initiated by nucleophilic species (OH^−^) existing in the aqueous medium, leading to rapid chain propagation and NP formation. Briefly, (0.25% *v*/*v*) ECA was added dropwise to 40 mL aqueous suspension of Mh NPs (0.125% *w*/*v*) adjusted to pH 4 under mechanical stirring (2000 rpm), using Polaxamer 188 (1% *w*/*v*) as a stabilizer to prevent aggregation. The reaction proceeded for 3 h. To optimize the size of the NPs, different Mh:PECA mass ratios (2:4, 2:3, 4:2, 3:2, and 2:2) were evaluated based on hydrodynamic diameter, polydispersity index (PdI), and *ζ*-potential. The initially described amounts of monomer and MNPs correspond to the 2:4 ratio, and variations in these quantities were subsequently performed to obtain the remaining formulations. Each formulation was prepared in triplicate.

Following this, Cs surface functionalization was achieved by coacervation, driven by electrostatic interactions between the positively charged amino groups of chitosan and the negatively charged surface of the Mh@PECA (core@shell) NPs, as previously described [31,32,33]. For this purpose, Poloxamer 188 (1% *w*/*v*) was added to a 25 mL aqueous acetic acid solution (2% *v*/*v*) containing Mh@PECA NPs (0.084% *w*/*v*), followed by the incorporation of Cs (0.042% *w*/*v*). Subsequently, 6.25 mL of a Na_2_SO_4_ solution (20% *w*/*v*) was slowly added under ultrasonication (20% amplitude, 40% pulse mode) for 15 min in an ice bath to maintain low temperature. Variations in the (Mh@PECA):Cs mass ratio were carried out based on the initially described proportion (4:2), resulting in additional formulations with ratios of 2:4, 2:3, 3:2, and 2:2. Each formulation was prepared in triplicate, and results are reported as mean values ± standard deviation (SD).

To identify the optimal formulations for subsequent characterization, the different Mh:PECA and Mh@PECA:Cs mass ratios were compared based on their hydrodynamic diameter, PdI, and *ζ*-potential. Formulations exhibiting the most favorable combination of these physicochemical parameters, indicative of controlled shell formation and satisfactory colloidal behavior, were selected for further physicochemical, magnetic, relaxometric, and biological characterization. This optimization strategy has been widely employed for the development of polymer- and polysaccharide-coated iron oxide nanostructures [34,35,36,37,38].

#### 2.2.2. Hydrodynamic Size, Polydispersity Index, and Zeta Potential

The hydrodynamic diameter, PdI, and zeta potential (*ζ*) of aqueous NP dispersions (0.1%, *w*/*v*) were measured at room temperature using a Zetasizer Nano ZS (Malvern Instruments Ltd., Worcestershire, UK). Measurements were performed in triplicate. Particle size and PdI were determined by photon correlation spectroscopy, while *ζ* was measured by electrophoretic light scattering at a detection angle of 60°.

The *ζ* of the NPs was evaluated for both Mh@PECA and (Mh@PECA)@Cs systems by analyzing 1 mM KNO_3_ aqueous NP dispersions (0.1%, *w*/*v*), over a pH range of 4 to 8. Measurements were performed at room temperature using a Zetasizer Nano ZS (Malvern Instruments Ltd., Worcestershire, UK) (*n* = 9).

#### 2.2.3. Colloidal Stability

The colloidal stability of Mh@PECA and (Mh@PECA)@Cs NPs was evaluated under both storage and accelerated aging conditions. For the long-term storage study, NP suspensions were stored at 4.0 ± 0.5 °C and 25.0 ± 0.5 °C for 25 days. At predetermined time intervals, the hydrodynamic diameter, PdI, *ζ*-potential, and pH were determined to monitor possible physicochemical changes during storage.

In addition, an accelerated stability study was performed using (Mh@PECA)@Cs NPs dispersed in phosphate-buffered saline (PBS; pH 7.0). Suspensions were maintained at 25.0 ± 0.5 °C and 72.0 ± 0.5 °C for predetermined time intervals. The elevated temperature (72 °C) was selected as an accelerated aging condition to promote potential destabilization processes, including particle aggregation, polymer degradation, and changes in surface properties, thereby enabling rapid evaluation of formulation stability. At each sampling time, the hydrodynamic diameter, PdI, and *ζ*-potential were determined by dynamic light scattering (DLS) using the experimental conditions described in Section 2.2.2. The pH was not monitored during this assay because the NPs were dispersed in PBS (initial pH 7.0), whose buffering capacity maintains a stable pH throughout the experimental period. All measurements were performed in triplicate (*n* = 3), and the results are reported as means ± standard deviation (SD).

#### 2.2.4. Transmission Electron Microscopy (TEM)

NP structures were examined by TEM using a LIBRA 120 PLUS (Carl Zeiss SMT GmbH, Oberkochen, Germany) microscope operating at an accelerating voltage of 120 kV. Samples were prepared by depositing a drop of NP suspension (0.1% *p*/*v*) onto carbon-coated copper.

The elemental composition and spatial distribution of the polymer coating were further investigated by high-angle annular dark-field scanning Transmission Electron Microscopy (HAADF-STEM) coupled with energy-dispersive X-ray spectroscopy (EDX) using a Talos F200X S/TEM microscope (Thermo Fisher Scientific, Waltham, MA, USA) operated at an accelerating voltage of 200 kV. Elemental mapping and line-scan analyses were performed to determine the distribution of Fe, N, and O within the nanostructures. In particular, HAADF-STEM–EDX line profiles were acquired across selected NPs to evaluate the spatial correlation between Fe, associated with the magnetosome core, and N, characteristic of the chitosan-containing polymeric coating, thereby providing evidence of the core–shell architecture of the (Mh@PECA)@Cs NPs.

#### 2.2.5. Fourier Transform Infrared Spectroscopy (FTIR) Analysis

Chemical characterization of the (Mh@PECA)@Cs NPs was performed by FTIR spectroscopy using an FT/IR-6200 spectrometer (JASCO Inc., Easton, MD, USA) at a resolution of 0.25 cm^−1^. Spectra were smoothed using a Savitzky–Golay filter (polynomial order 2, 11-point window) to reduce noise without distorting spectral features.

#### 2.2.6. Magnetic Characterization

The magnetic properties of the (Mh@PECA)@Cs NPs were evaluated and compared to Mh and Mh@PECA NPs by recording hysteresis loops between 0 and 4000 kA/m using a vibrating sample magnetometer (VSM; DSM-8, Manics, Ernolsheim-Bruche, France) at room temperature.

The initial magnetic susceptibility (*χ*_0_) was obtained from the hysteresis loops by calculating the slope of magnetization as a function of the applied magnetic field (H) in the low-field region near H = 0. A linear fit was applied to the initial segment of the magnetization curve to determine *χ*_0_, assuming a linear response in this region. The saturation magnetization (*M*_s_) was determined from the hysteresis loops as the magnetization value reached at high H, where further increases in the field produced no significant changes in magnetization. All measurements were performed in triplicate, and the results were expressed as mean values ± standard deviation.

#### 2.2.7. Iron Content Determination

The iron content of the NPs was quantified by inductively coupled plasma high-resolution mass spectrometry (ICP-HRMS) using an Element XR system (Thermo Fisher Scientific Inc., Bremen, Germany). Results were cross-validated by inductively coupled plasma atomic emission spectroscopy to ensure analytical accuracy.

#### 2.2.8. Cell Culture and Viability

Cells of the HeLa cell line were obtained from the American Type Culture Collection (ATCC, CCL-2; Manassas, VA, USA). A total of 10^6^ cells were cultured in DMEM and maintained at 37 °C in a humidified atmosphere containing 5% CO_2_.

Cell viability was evaluated using the trypan blue exclusion assay after 24 and 48 h of exposure to Mh@PECA and (Mh@PECA)@Cs NPs at concentrations ranging from 0 to 100 µg/mL. Cells were stained with 10% trypan blue, and live and dead cells were manually counted using a Neubauer chamber under a light microscope. Results were expressed as mean values ± SD (*n* = 3).

#### 2.2.9. MRI

##### Evaluation of Imaging Capability Based on Relaxometric Analysis

Longitudinal (*r*_1_) and transverse (*r*_2_) relaxivity of the Mh@PECA and (Mh@PECA)@Cs NPs were determined at 37.0 ± 0.5 °C using a Bruker Minispec MQ-60 spectrometer (Bruker BioSpin, Rheinstetten, Germany) working at 1.41 T, which is the standard magnetic field for quantitative relaxometric characterization with this instrument and is close to the clinically established 1.5 T field strength. In contrast, phantom MRI experiments were performed with a clinical scanner at 3 T, another widely used field strength in clinical practice. Since the relaxometric performance of SPION-based contrast agents is magnetic field-dependent, characterization at both 1.41 T and 3 T provides complementary information regarding the MRI performance of the proposed nanocomposites under clinically relevant conditions.

Longitudinal (*T*_1_) and transverse (*T*_2_) relaxation times were determined using the inversion recovery and Carr–Purcell–Meiboom–Gill (CPMG) sequence.

Relaxivity was calculated according to the linear relationships in Equation (1):

(1) $$\frac{1}{T_{i}}=\frac{1}{T_{i,0}}+r_{i}[Fe]$$

where *i* corresponds to either longitudinal (*i* = 1) or transverse (*i* = 2) relaxation, $T_{i}$ is measured relaxation time, *T_i_*_,0_ is the corresponding relaxation time of the solvent in the absence of NPs, [*Fe*] is the iron concentration (mM), and *r_i_* is the corresponding longitudinal (*r*_1_) or transverse (*r*_2_) relaxivity (mM^−1^·s^−1^).

Relaxometry measurements were performed in triplicate using iron concentrations from 45 to 300 μM. Longitudinal and transverse relaxivity were calculated individually from the slopes of three independent linear regressions of 1/*T*_1_ and 1/*T*_2_ versus Fe concentration, respectively, each corresponding to one experimental replicate. The reported relaxivity values represent the means ± standard deviation of these independent determinations.

##### Acquisition of Magnetic Resonance Imaging Phantom Images

MRI experiments were performed using a 3 T horizontal-bore benchtop MRI system (MR Solutions, Ltd., Guildford, UK) equipped with actively shielded gradients (48 G·cm^−1^) and a 56 mm quadrature birdcage coil operating in transmit/receive mode.

For phantom studies, aqueous NP suspensions with iron concentrations ranging from 45 to 300 μM were prepared (300 μL) and loaded into a custom 3D-printed polylactic acid well plate. Parametric *T*_2_ maps were acquired using MEMS (multi-echo multi-slice) sequences with the following parameters: *TE* (echo time) = 64 ms, *TR* (repetition time) = 5000 ms, *NE* (number of echoes) = 15, *N_S_* (number of slices) = 3, *S*_T_ (slice thickness) = 1 mm, and *AT* (acquisition time) = 32 min. All MR images were acquired with an image matrix 256 × 252, *FOV* (Field of View) 60 × 60 mm, *NA* (number of averages) = 15. Fiji (ImageJ version 1.54p; National Institutes of Health, Bethesda, MD, USA) software was used to reconstruct the maps using the “MRI analysis calculator” plugin by Karl Schmidt.

##### Cellular MRI Visualization of NPs Internalized in HeLa Cells

Cellular phantoms were generated by incubating 10^6^ HeLa cells with NP suspensions at iron concentrations of 0, 10, 50, and 100 μM for 48 h. After incubation, cells were washed to remove non-internalized NPs, trypsinized, and centrifuged (1200 rpm, 5 min) to obtain cell pellets. The pellets were resuspended in 1% (*w*/*v*) agar to form homogeneous gels, which were subsequently transferred into 300 μL tubes for imaging.

*T*_2_-weighted images were acquired at 3.0 T using FSE (fast spin echo sequences) with the following parameters: *TE* = 11 ms, *TR* = 12,000 ms, *NA* = 10, *N_S_* = 3, *ST* = 1 mm, and *AT* = 20 min. Signal intensity values were extracted from regions of interest (ROIs) using FIJI software and used for relative comparison between samples.

#### 2.2.10. HeLa Cell Internalization Capacity Determined by Iron Quantification

HeLa cells (10^6^ cells) were incubated in DMEM for 24 h. Then, cells were exposed to 100 µg/mL (Mh@PECA)@Cs NPs for 24 h. A control group of HeLa cells without NP exposure was processed under identical conditions.

After incubation, the supernatant containing non-internalized NPs was removed, and cells were washed twice with HBSS. Cells were then collected and centrifuged (1200 rpm, 5 min) to obtain the final cell pellet, and the supernatant was carefully aspired.

For sample preparation, 1 mL of 37% HCl was directly added to the cell pellet to ensure complete digestion of cellular and organic material. The samples were subsequently diluted in 9 mL of water.

The intracellular iron content, indicative of NP internalization, was quantified by ICP-HRMS analysis. All experiments were performed in triplicate, and the results are represented as mean values ± SD.

#### 2.2.11. Evaluation of Hyperthermia-Induced Effects in HeLa Cells

HeLa cells (5 × 10^5^ cells per dish) were seeded in 35 mm culture dishes and incubated in DMEM for 24 h. Cells were then treated with Mh@PECA or (Mh@PECA)@Cs NPs at concentrations of up to 100 µg/mL for 5 h. Then, the cultures were exposed to an AMF using a live-cell magnetic hyperthermia system (NanoTherics Ltd., Warrington, UK) at 37.0 °C ± 0.5 °C and under 5% CO_2_ atmosphere. Hyperthermia treatment was performed at a frequency of 280 kHz and a magnetic field amplitude of 20 mT for 1 h.

Then, 48 h post-treatment, cells were washed twice with HBSS, and cell viability was assessed using the trypan blue exclusion assay. Cell viability values were normalized to the control group (untreated cells), which was set to 100%. Statistical analysis was carried out using Student’s *t*-test, considering *p* < 0.05 as the threshold for statistical significance.

These biological experiments were designed as a preliminary proof of concept to evaluate whether the nanosystem could induce a measurable hyperthermia-mediated cytotoxic effect in a representative tumor cell line. HeLa cells were selected because they constitute one of the most widely used cancer models in magnetic hyperthermia research, enabling direct comparison with previously reported MNP systems [26,39,40,41,42].

#### 2.2.12. Heating Profile

The evaluation of heat generation was performed with a DM 100 System (nB nanoScale Biomagnetics S.L., Zaragoza, Spain) by acquiring magnetic heating curves. Measurements were performed using 500 μL aliquotsof aqueous suspensions formulations of the Mh and Mh@PECA NPs (Fe = 2.3 g/L) and (Mh@PECA)@Cs NPs (Fe = 0.23 g/L). Each aliquot was transferred into a 1.5 mL glass vials and were placed at the midpoint of a water-cooled copper coil. Then AMF was applied: 869 kHz and 20 mT. These AMF parameters were selected because they correspond to the maximum frequency and magnetic field amplitude available in the experimental setup, thereby maximizing the temperature increase and enabling accurate characterization of the intrinsic heating performance of the NPs. These conditions were used exclusively for physicochemical characterization and differ from those employed in the in vitro hyperthermia experiments (Section 2.2.10). The temperature was monitored as a function of time while applying the AMF by an integrated optical fiber-based temperature measurement system.

For magnetic hyperthermia characterization, the highest frequency and magnetic field amplitude available in the experimental setup were selected to maximize the temperature increase and allow for accurate determination of the heating performance of the different formulations. These experimental conditions were employed exclusively for in vitro characterization of the magnetic heating properties and do not necessarily correspond to the AMF parameters intended for future clinical applications [43,44].

Specific absorption rates (SARs; W/g Fe) were calculated according to Equation (2):

(2) $$SAR=\frac{C_{water}}{\left[Fe\right]}\;\cdot \frac{dT}{dt}$$

where C is the specific heat capacity of the medium (assumed equal to that of water, C_water_ = 4185 J·L^−1^·K^−1^), [Fe] is the iron concentration (gFe·L^−1^) of the magnetic material in solution, and dT/dt is the slope of the initial linear section of the temperature-versus-time curve. The SAR values were determined by the initial slope method. For the calculations, the linear region of the heating curves was defined within the 50–150 s time interval after the application of the AMF, and the slope was obtained by linear regression analysis.

The intrinsic loss power (ILP) was calculated from the experimentally determined SAR values according to Equation (3):

(3) $$ILP=\frac{SAR}{H^{2}f}$$

where *H* is the amplitude of the AMF expressed in A m^−1^, and *f* is its frequency expressed in Hz. In the present experiments, a magnetic flux density of 20 mT, corresponding to *H* = 15.9 kA m^−1^, and a frequency of 869 kHz were applied; ILP was expressed in nH m^2^ kg^−1^.

#### 2.2.13. Hemocompatibility Assays

The experimental protocol was adapted from previously established methodologies for the ex vivo evaluation of NP–blood interactions [45,46]. Blood samples were collected from two healthy female donors (24 and 45 years old) following approval by the Andalusian Biomedical Research Ethics Committee (Consejería de Salud de la Junta de Andalucía; 2020522131049). Blood was collected using EDTA-containing tubes (for hemolysis and platelet activation assays) and sodium citrate-containing tubes (for complement activation and coagulation studies). NP dispersions were incubated with blood aliquots to evaluate their interactions with erythrocytes, platelet activation, complement system response, and coagulation parameters. Each experimental condition was analyzed in triplicate (*n* = 3), following standard hemocompatibility assessment protocols [45].

Endpoint-specific negative and positive controls were processed in parallel with the NP-treated samples. PBS was used as the negative control in all assays. Triton X-100 (1% *v*/*v*), thrombin receptor-activating peptide 6 (TRAP-6; 50 µM), zymosan (1 mg/mL), and kaolin (1 mg/mL) were used as positive controls for hemolysis, platelet activation, complement activation, and coagulation, respectively.

For hemolysis assessment, erythrocytes were isolated from EDTA-treated blood by centrifugation at 2000 rpm for 5 min (two cycles), followed by incubation with NP suspensions (1:10 in PBS) for 2, 6, 12 and 24 h at 37.0 ± 0.5 °C. After incubation, samples were centrifuged (2000 rpm, 5 min), and hemoglobin release in the supernatant was quantified by measuring the optical density at 540 nm, using a validated UV–Vis spectrophotometric method with a LAMBDA™ 25 UV–Vis spectrophotometer (PerkinElmer Inc., Waltham, MA, USA) [46,47]. The percentage of hemolysis was normalized to the negative and positive controls according to Equation (4):

(4) $$Hemolysis\left(\%\right)=\frac{Asample-\;Anegative\;control}{Apositive\;control-Anegative\;control}\times 100$$

where *A*_sample_, *A*_negative control_, and *A*_positive control_ denote the absorbance values obtained for the NP-treated, PBS-treated and Triton X-100-treated erythrocytes, respectively. Accordingly, PBS and Triton X-100 represented 0% and 100% hemolysis, respectively.

Platelet activation was evaluated by quantifying soluble P-selectin (sP-selectin) levels in plasma using an enzyme-linked immunosorbent assay (ELISA), with PBS and TRAP-6 serving as the negative and positive controls, respectively. Complement activation was determined by quantifying plasma C3a-desArg levels using ELISA, with PBS as the negative control and zymosan as the positive control. The potential effect of the NPs on coagulation was evaluated by measuring the plasma recalcification time (PRT) according to the Howell method [45], using PBS and kaolin as the negative and positive controls, respectively.

## 3. Results and Discussion

### 3.1. Formulation Screening: Hydrodynamic Size, PdI, and Surface Electrical Charge

During formulation optimization, the mass ratios among components used to obtain the Mh@PECA and (Mh@PECA)@Cs NPs were systematically evaluated. Hydrodynamic size and PdI were used as primary criteria to identify formulations compatible with biomedical constraints (colloidal stability, biological interaction, and reproducibility).

For the Mh@PECA NPs, increasing the Mh proportion at constant ECA monomer content led to an increase in NP size. Conversely, when Mh content was kept constant and the monomer fraction was reduced, size changes were not linear (Table 1), suggesting a non-trivial dependence on the balance between amount of ECA and final particle size.

Based on the mass variation results of Mh:ECA (Table 1), the smallest NPs, corresponding to the (2:4) formulation, were selected for subsequent surface functionalization with Cs. Upon Cs incorporation, an increase in either the Mh@PECA NP proportion or the Cs amount resulted in larger NPs (Table 2). The formulation yielding the smallest NPs (4:2) exhibited a reduced PdI of ~0.2, indicating a narrower and more homogeneous size distribution. This value falls within the generally accepted range for biomedical applications (PdI ≤ 0.3), which is associated with improved colloidal stability and reproducible nano–bio-interactions, both critical to predictable cellular uptake and in vivo performance [48,49].

**Figure 1:**
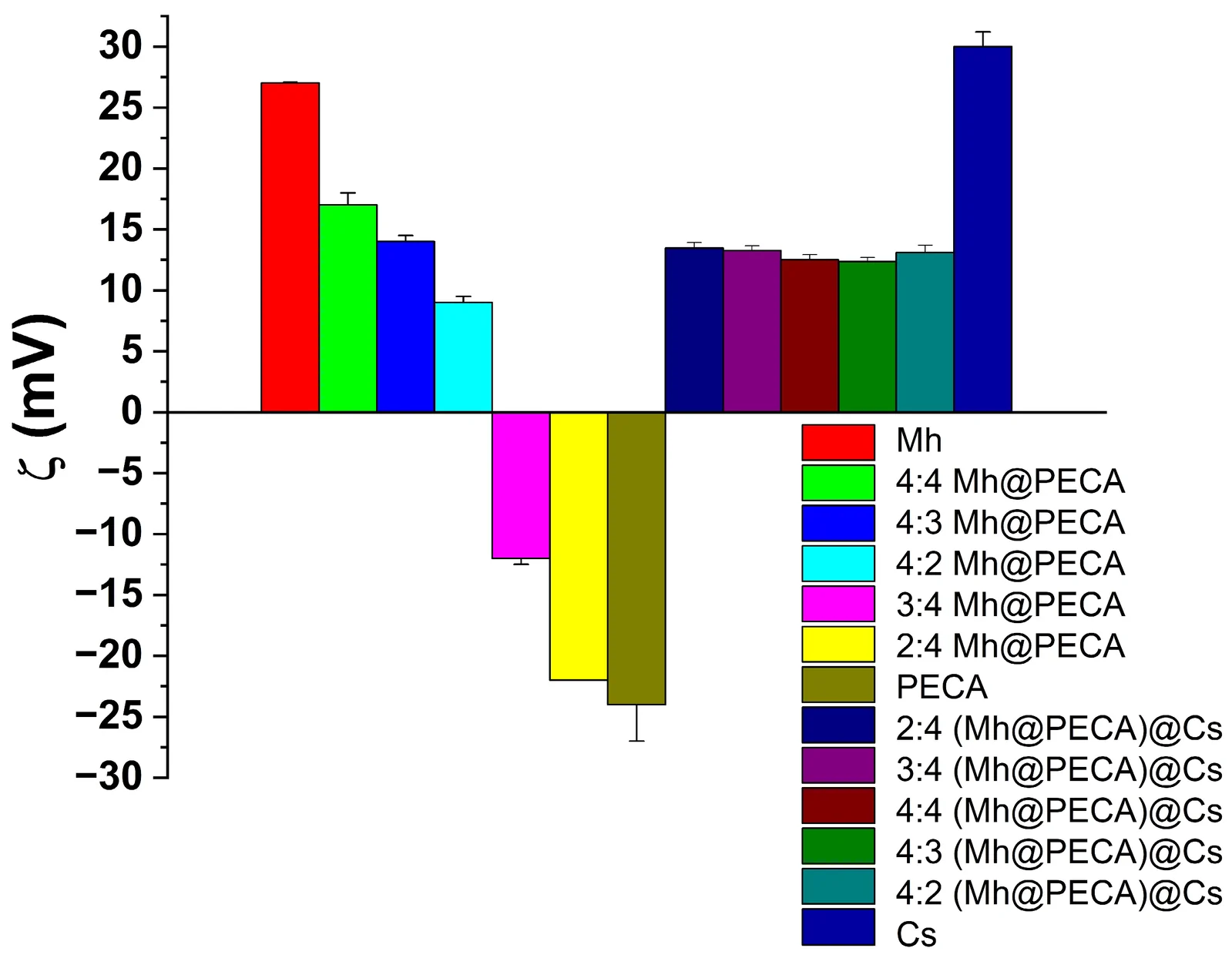
*ζ* (mV) of Mh, Mh@PECA, and (Mh@PECA)@Cs NPs as a function of the Mh-to-polymer mass ratio. Mh@PECA formulations exhibit a progressive shift towards negative surface charge with increasing polymer content, approaching values characteristic of PECA, whereas (Mh@PECA)@Cs systems display positive *ζ*-potential values across all tested ratios, consistent with Cs surface functionalization. Data are presented as mean values ± standard deviation (*n* = 3).

The evaluation of the surface electrical charge confirmed the presence of the polymers in the outermost layer of the NPs. In the case of Mh@PECA NPs, only the 2:4 and 3:4 ratios exhibited a negative surface charge, as expected for PECA due to the presence of carboxylate (–COO^−^) groups. Among them, the 2:4 ratio showed values closer to those of pure PECA NPs, as shown in Figure 1. Regarding the (Mh@PECA)@Cs NP formulations, all tested ratios exhibited positive *ζ* values, like those obtained for Cs NPs.

Based on the results in Table 1 and Table 2 and Figure 1, the formulations selected were those with the 2:4 Mh:PECA mass ratio and 4:2 (Mh@PECA):Cs mass ratio, which were characterized by smaller particle sizes (228.81 nm and 304.05 nm, respectively) while maintaining acceptable PdI values (0.334 and 0.215, respectively) [32,33]. In addition, the Mh@PECA NPs prepared in a 2:4 ratio displayed a surface charge more closely resembling that of pure PECA NPs, indicating more effective polymer coating compared with the other tested ratios.

In contrast, all (Mh@PECA)@Cs formulations exhibited positive *ζ*-potential values, consistent with the presence of Cs at the NP surface and a clear shift away from the negative charge associated with PECA. Since the surface charge was similar across all tested ratios, the selection of the optimal (Mh@PECA):Cs formulation was primarily based on particle size and PdI, with the 4:2 ratio providing the smallest NPs while maintaining acceptable dispersity. The positive *ζ*-potential further confirms the successful surface functionalization with Cs. These physicochemical characteristics were therefore considered the most suitable for subsequent characterization.

### 3.2. Physicochemical Characterization

The colloidal stability of Mh@PECA and (Mh@PECA)@Cs NPs was evaluated over a 25-day period by monitoring their hydrodynamic diameter, PdI, *ζ*, and pH under storage at 4.0 ± 0.5 °C and 25.0 ± 0.5 °C (Table 3).

(Mh@PECA)@Cs NPs retained a positive *ζ* throughout the study, indicating that the chitosan coating remained stable during storage. No significant variations in hydrodynamic diameter were observed over the 25-day period (348.40 ± 47.76 nm on day 0 and 394.28 ± 20.67 nm after 25 days), demonstrating good colloidal stability. The PdI values fluctuated throughout the storage period at both temperatures, with no evidence of a progressive increase indicative of particle aggregation. Overall, these results, together with the hydrodynamic diameter and *ζ*-potential measurements, support the preservation of the colloidal stability of the NP suspensions during storage. Likewise, the pH remained essentially constant throughout the storage period under both temperature conditions, indicating that no appreciable changes in the physicochemical environment of the suspensions occurred during the study.

Although the selected Mh@PECA formulation exhibited an initial PdI of 0.321 ± 0.02, the colloidal stability study demonstrated that this degree of particle size heterogeneity did not compromise the physicochemical stability of the nanosystem during the 25-day storage period. Despite the fluctuations observed in PdI, no progressive increase in particle size or loss of surface charge was detected, and the pH remained stable throughout the study, indicating that the NPs maintained their colloidal integrity under the evaluated storage conditions.

Nevertheless, particle size distribution remains an important design parameter for nanomedicines, as it may influence colloidal behavior, protein corona formation, cellular uptake, biodistribution, and pharmacokinetics. Reducing particle size heterogeneity could further improve the uniformity of NP interactions with biological systems, thereby enhancing batch-to-batch reproducibility and facilitating the translational development of the proposed nanoplatform. In addition, broader size distributions may promote the coexistence of particles that follow distinct endocytic routes or exhibit different colloidal stabilities, potentially leading to heterogeneous intracellular processing, variable biological responses, and increased experimental variability [50,51,52]. Moreover, the hydrodynamic diameters of both NP types (230–290 nm for Mh@PECA and 348–464 nm for (Mh@PECA)@Cs) fall within a size regime that is commonly associated with reduced renal clearance, limited susceptibility to nonspecific protein adsorption, and preferential uptake through endocytic pathways compatible with efficient intracellular processing. NPs in this size range can also interact with biological barriers in a more predictable manner, which mitigates the functional impact of moderate polydispersity and supports the overall stability and biological performance of the platform [50,51,52,53].

The colloidal stability of (Mh@PECA)@Cs NPs was further evaluated during incubation in PBS (pH 7.0) at 25.0 ± 0.5 °C and 37.0 ± 0.5 °C for up to 120 h (Table 4). Overall, the formulation maintained its colloidal stability under both incubation conditions, with only minor variations in hydrodynamic diameter, PdI, and *ζ*-potential throughout the study.

At 25.0 °C, the hydrodynamic diameter remained within a relatively narrow range throughout the incubation period, while the PdI fluctuated without showing a progressive increase indicative of particle aggregation. Likewise, the *ζ* exhibited only a slight decrease over time while remaining positive, indicating that the surface characteristics of the NPs were preserved during incubation.

A similar behavior was observed at 37.0 °C. Although slightly higher hydrodynamic diameter and PdI values were recorded after 24 h, both parameters fluctuated during the incubation period without evidence of progressive particle growth or colloidal destabilization. The *ζ*-potential also decreased slightly but remained positive throughout the experiment, indicating that the chitosan-coated NPs preserved their surface charge under physiological temperature.

Overall, the combined analysis of hydrodynamic diameter, PdI, and *ζ*-potential indicates that incubation in PBS at both 25.0 °C and 37.0 °C did not induce significant colloidal destabilization over the evaluated period. Although minor fluctuations in these physicochemical parameters were observed, no consistent trend associated with irreversible aggregation or loss of colloidal integrity was detected. These findings support the physicochemical stability of the formulation under conditions relevant for subsequent biological evaluation.

#### 3.2.1. Effect of pH on the Electrokinetics

**Figure 2:**
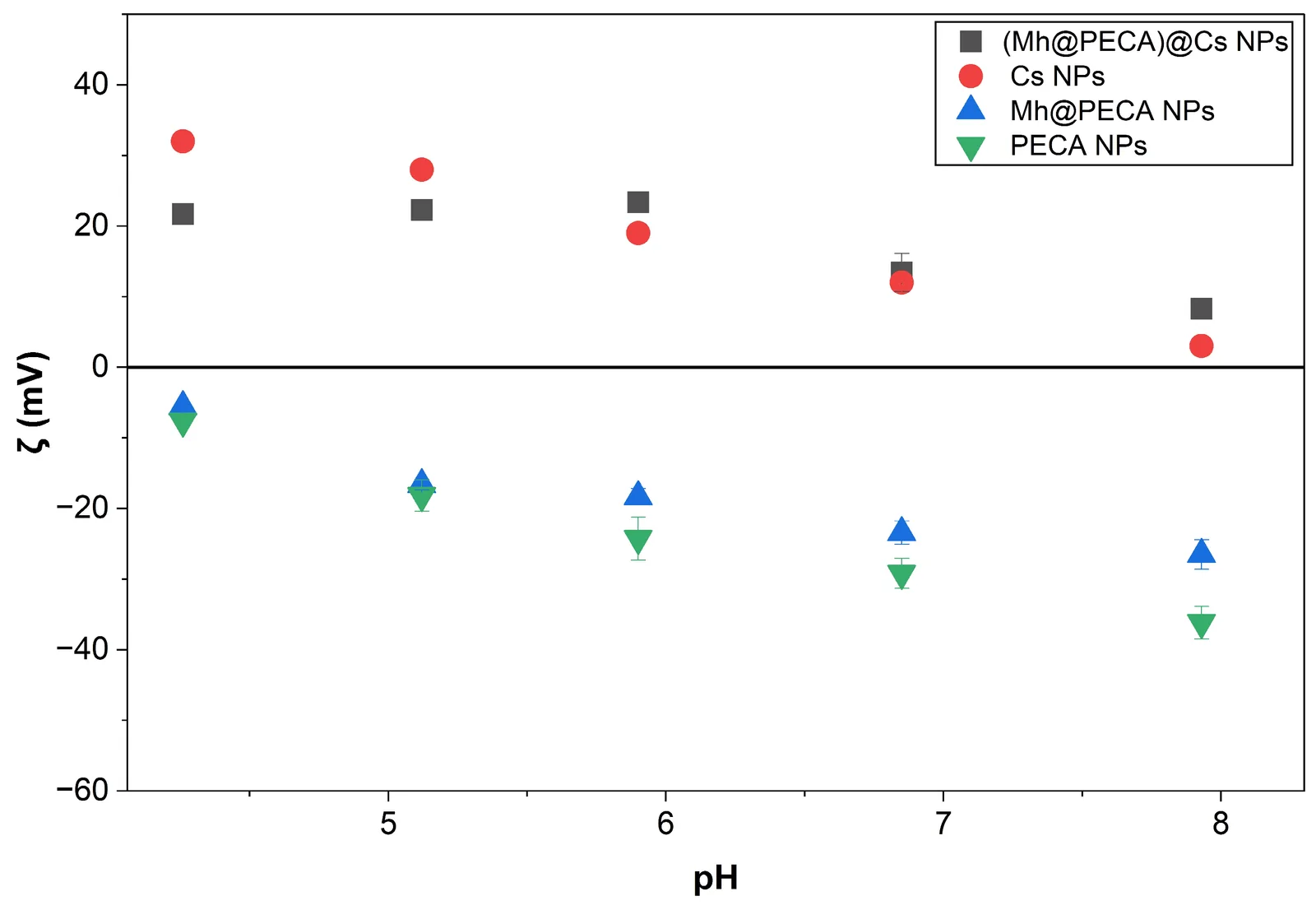
*ζ* (mV) as a function of pH for Cs, PECA, Mh@PECA, and (Mh@PECA)@Cs NPs in the presence of 1 mM KNO_3_. The surface charge evolution reflects the contribution of each component to the NP interface, with Cs-containing systems exhibiting positive values over a wide pH range, while PECA-based systems show predominantly negative *ζ*-potential. Data are expressed as means values ± SD (*n* = 3).

Surface functionalization of the Mh NPs by the polymers was supported by electrokinetic analysis. When the *ζ*-potential was evaluated as a function of pH (Figure 2), the Mh@PECA NPs exhibited negative *ζ*-potential values across the entire pH range studied. These results were like those obtained for PECA NPs and clearly differed from those of Mh NPs, which exhibited positive surface charge values.

In contrast, (Mh@PECA)@Cs NPs displayed markedly different *ζ* values compared with Mh@PECA NPs, showing positive *ζ* values across the studied pH range, similar to those observed for pure Cs NPs.

#### 3.2.2. FTIR Analysis

**Figure 3:**
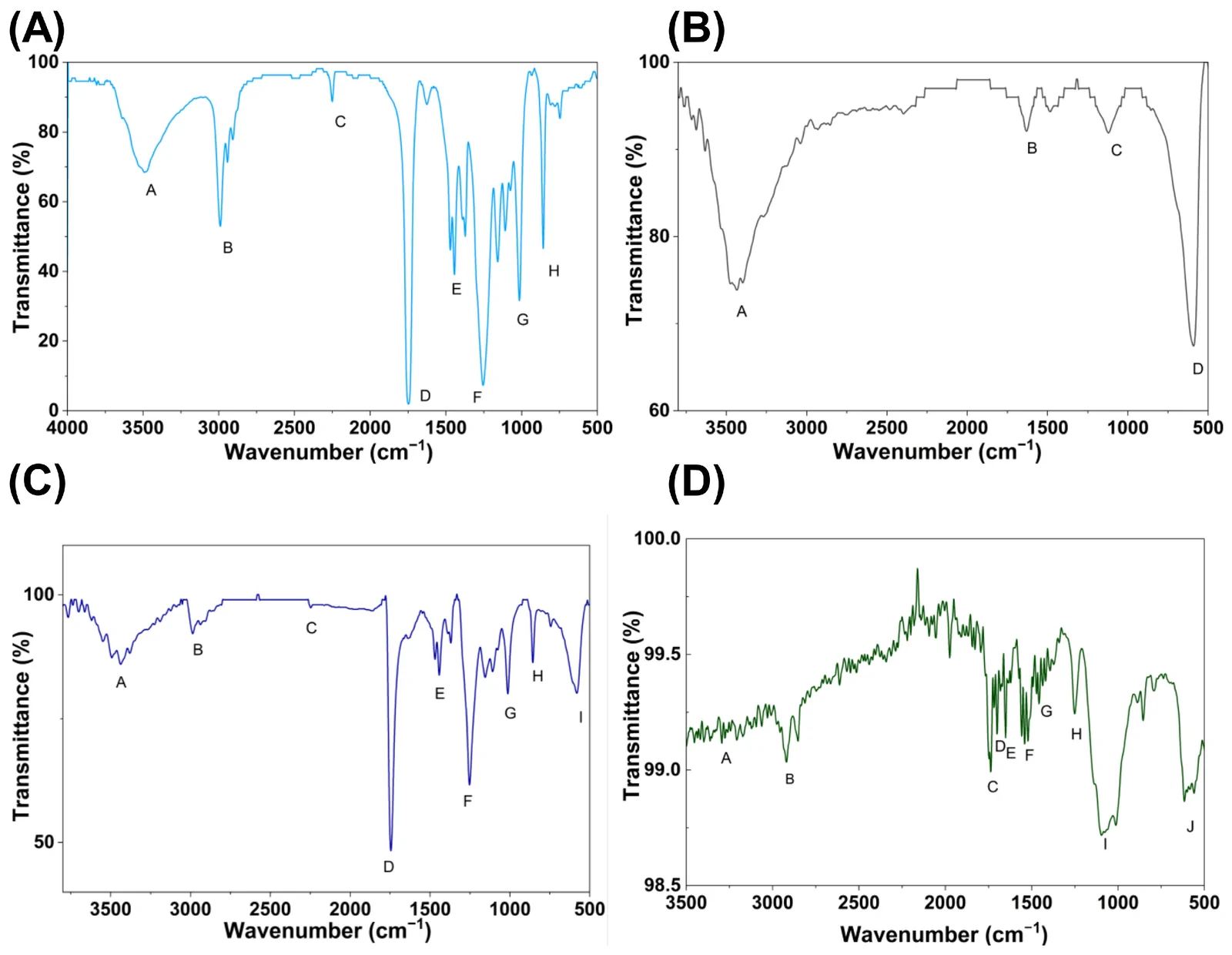
FTIR spectrum of the PECA NPs (**A**), Mh NPs (**B**), Mh@PECA (**C**) and (Mh@PECA)@Cs hybrid NPs (**D**), showing characteristic bands of Cs, PECA, and Mh.

The FTIR spectra of PECA, Mh, Mh@PECA, and (Mh@PECA)@Cs (Figure 3A–D) confirm the presence of each component and the successful formation of the hybrid systems, with all labeled bands (A–J) consistently assigned [54].

The PECA spectrum (Figure 3A) shows a broad band at ~3400 cm^−1^ (A) attributed to O–H stretching from adsorbed water. Bands at ~2990–2950 cm^−1^ (B) correspond to aliphatic C–H stretching, while a weak signal at ~2250–2200 cm^−1^ (C) is assigned to the nitrile group. The strong band at ~1740–1720 cm^−1^ (D) corresponds to ester C=O stretching. Additional bands at ~1450–1380 cm^−1^ (E) arise from C–H bending, whereas the regions at ~1300–1150 cm^−1^ (F) and ~1100–1000 cm^−1^ (G) are assigned to C–O–C and C–O stretching vibrations. Weaker bands at ~900–750 cm^−1^ (H) correspond to skeletal vibrations of the polymer.

The Mh spectrum (Figure 3B) presents bands at ~3400 cm^−1^ (A) and ~1600 cm^−1^ (B), attributed to the O–H stretching and H–O–H bending of adsorbed water. Weak contributions at ~1100–1000 cm^−1^ (C) may be related to surface Fe–OH groups. The strong band at ~580–550 cm^−1^ (D) corresponds to Fe–O stretching vibrations, confirming the iron oxide lattice.

In the Mh@PECA spectrum (Figure 3C), the coexistence of both components is evidenced by the O–H band (A), C–H stretching (B), and a weak or poorly resolved nitrile contribution (C). The ester C=O band at ~1730 cm^−1^ (D) and C–H deformation modes (F) are clearly observed, together with the intense C–O–C/C–O region (~1300–1000 cm^−1^; G). The H–O–H bending at ~1600 cm^−1^ (E) remains visible. The band near 900 cm^−1^ (H) may be attributed to out-of-plane =C–H bending or terminal/residual unsaturated groups. Importantly, the Fe–O band at ~580–600 cm^−1^ (I) confirms that the iron oxide core is preserved after coating.

Finally, the (Mh@PECA)@Cs spectrum (Figure 3D) shows additional contributions from chitosan. The broad band at ~3200–3500 cm^−1^ (A) corresponds to overlapping O–H/N–H stretching vibrations. Bands at ~2920–2850 cm^−1^ (B) are assigned to aliphatic C–H stretching, while the ester C=O (~1750–1725 cm^−1^; C) remains present. The bands at ~1655–1630 cm^−1^ (D) and ~1595–1540 cm^−1^ (E) correspond to amide I/bound water and N–H bending (amide II), respectively. The region at ~1450–1400 cm^−1^ (F) is assigned to CH_2_/CH_3_ bending, and the band at ~1320–1250 cm^−1^ (G) to C–N or amide III vibrations. The region at ~1240–1150 cm^−1^ (H) corresponds to C–O–C/C–O stretching, while the band at ~1100–1020 cm^−1^ (I) is associated with glycosidic C–O vibrations. Finally, the Fe–O band at ~630–550 cm^−1^ (J) confirms the presence of the iron oxide core.

Overall, the FTIR results confirm the successful formation of the Mh@PECA and (Mh@PECA)@Cs hybrid systems, preserving the iron oxide core while incorporating the polymeric components.

#### 3.2.3. TEM Study

**Figure 4:**
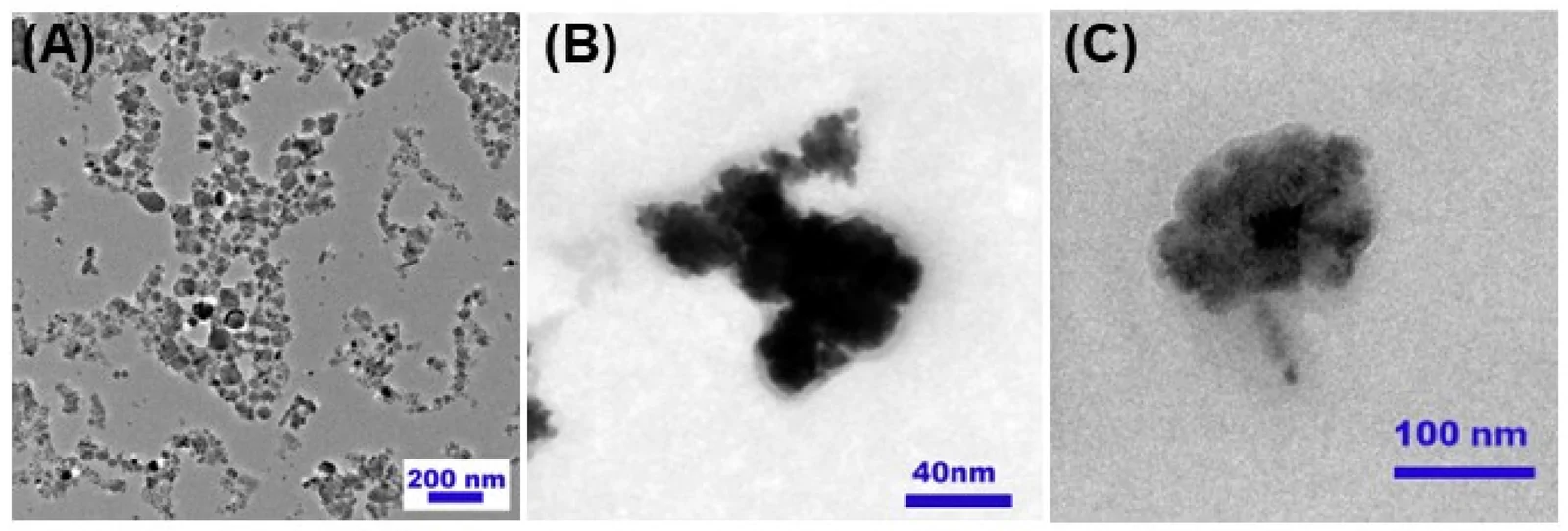
TEM images of (**A**) Mh NPs, (**B**) Mh@PECA NPs, and (**C**) (Mh@PECA)@Cs NPs.

The images revealed that the magnetic cores (Figure 4A) were completely covered by the polymeric material (Figure 4B,C), with highly electron-dense regions detected within the inner core of the platform.

**Figure 5:**
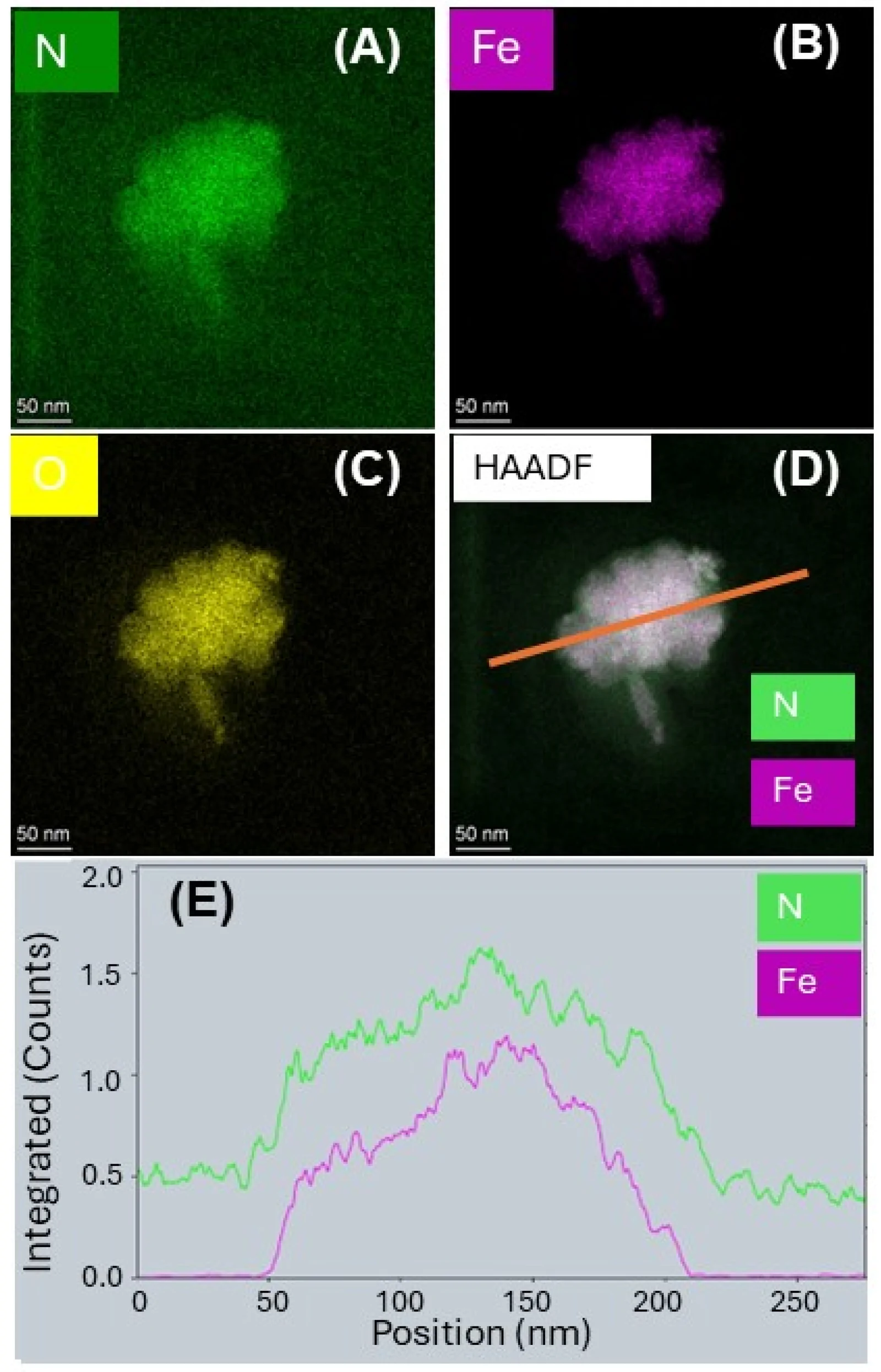
EDX elemental maps showing the spatial distribution of N (**A**), Fe (**B**), and O (**C**) in (Mh@PECA)@Cs NPs. Corresponding HAADF-STEM image (**D**) and elemental line-scan intensity profiles (**E**) for N and Fe extracted along the orange line marked in (**D**).

The EDX elemental maps (Figure 5) revealed the spatial distribution of Fe, O, and N within the (Mh@PECA)@Cs NPs. The Fe and O signals (Figure 5B,C) were homogeneously distributed throughout the NP, confirming the uniform dispersion of the Mh phase within the NPs. In contrast, the N signal (Figure 5A), which can be attributed to the polymeric components of the NPs, was distributed across the nanocomposites and extended over regions broader than those defined by Fe. Furthermore, the HAADF-STEM image (Figure 5D) together with the corresponding elemental line-scan profiles (Figure 5E) showed that the nitrogen signal remained spatially associated with the NPs while extending beyond the Fe-rich region. This distribution is consistent with the presence of a polymeric layer surrounding the magnetic phase rather than being confined to it. Although EDX does not permit direct measurement of the coating thickness, these complementary analyses provide strong structural evidence supporting the proposed (core@shell)@shell architecture of the (Mh@PECA)@Cs nanocomposites.

#### 3.2.4. Magnetic Characterization

**Figure 6:**
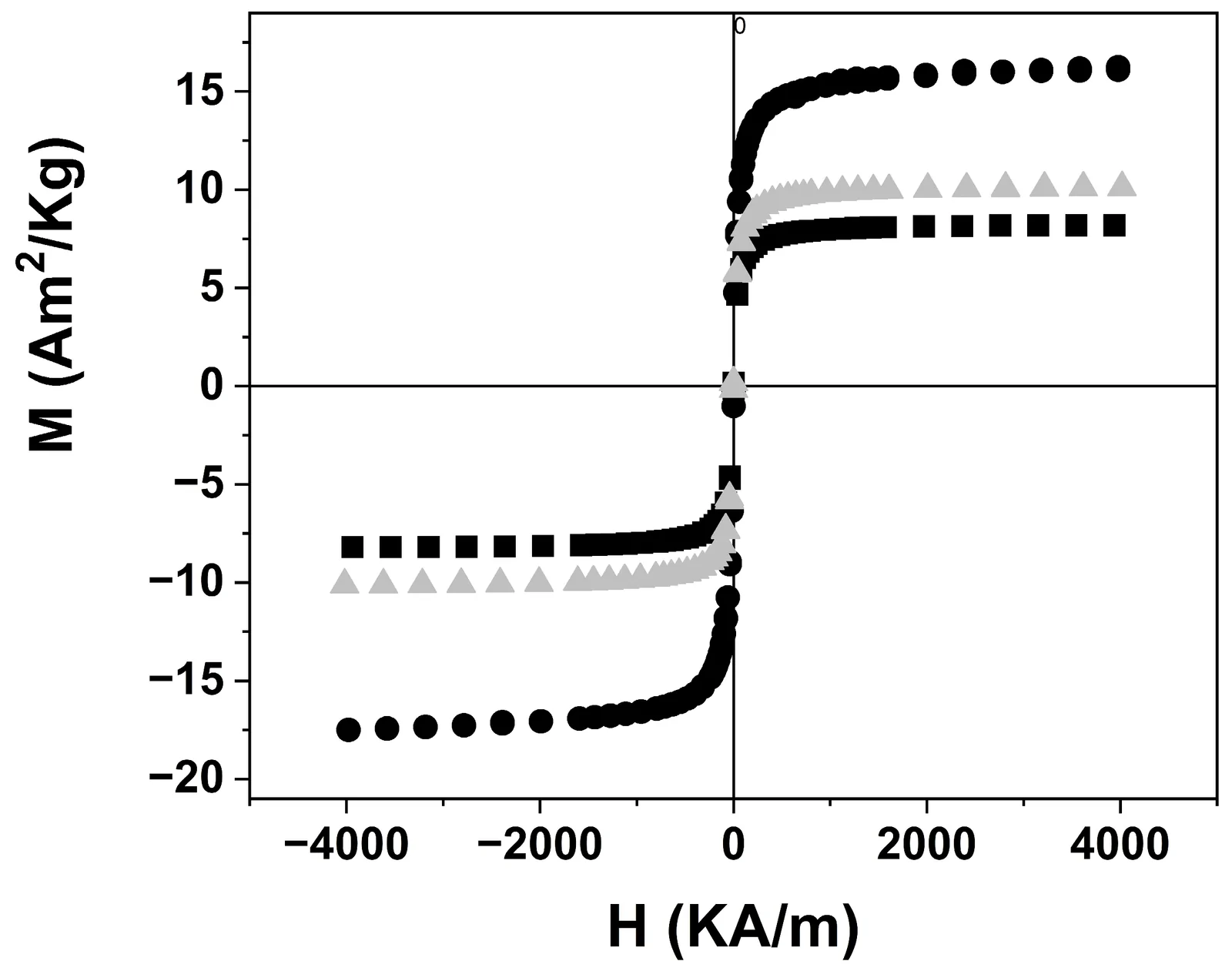
Hysteresis cycles of MNPs: Mh@PECA (▲), (Mh@PECA)@Cs (■), and Mh (●).

Magnetic characterization confirmed a superparamagnetic-like behavior as evidenced by sigmoidal hysteresis loops with negligible coercivity and remanence (Figure 6). These magnetic profiles of the NPs were consistent with superparamagnetic behavior at room temperature, which is critical to biomedical applications, particularly MRI contrast enhancement and magnetic hyperthermia [55,56,57]. In this context, SPIONs exhibit magnetization only under an external magnetic field and no remanence once the field is removed, preventing irreversible aggregation while preserving their effectiveness as MRI contrast agents and enabling controlled performance in applications such as magnetic targeting and hyperthermia [56,57,58].

The decrease in both *χ*_0_ and *M*_s_ in Mh@PECA and (Mh@PECA)@Cs NPs, compared with bare Mh cores (Table 5), can be primarily attributed to the presence of a magnetically inactive polymeric shell. This coating introduces a mass dilution effect, reducing the magnetic contribution per unit mass of the hybrid system [15,59,60].

In addition, the polymeric layer may induce surface spin disorder (spin canting) at the NP interface due to broken exchange interactions and reduced magnetic coupling at the surface. This phenomenon leads to a fraction of spins that do not align fully even under high magnetic fields, thereby decreasing the effective *M*_s_ [61,62].

Therefore, the reduction in *M*_s_ is therefore not indicative of a deterioration of the magnetic core itself but rather reflects the structural and compositional characteristics of the hybrid nanoplatform.

Preservation of superparamagnetic behavior of the Mh nuclei after PECA encapsulation and Cs functionalization was clear in Figure 6, supporting feasibility for theranostic use.

#### 3.2.5. Relaxometric Characterization and Imaging Capability Assessment

**Figure 7:**
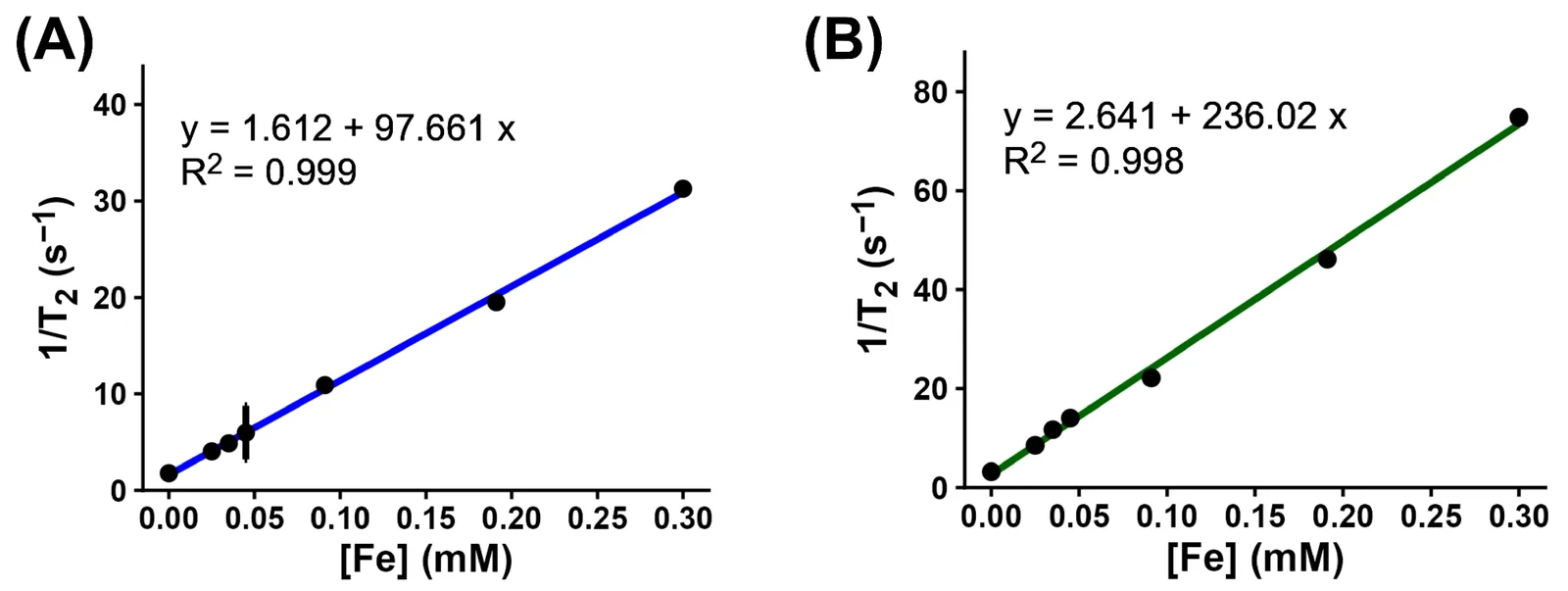
Transverse relaxation rates (1/*T*_2_, s^−1^) as a function of Fe concentration (mM) in (**A**) Mh@PECA and (**B**) (Mh@PECA)@Cs NPs, measured at 1.41 T. Black circles represent the experimental data, expressed as mean ± SD (*n* = 3), and solid lines represent the corresponding linear fits.

**Figure 8:**
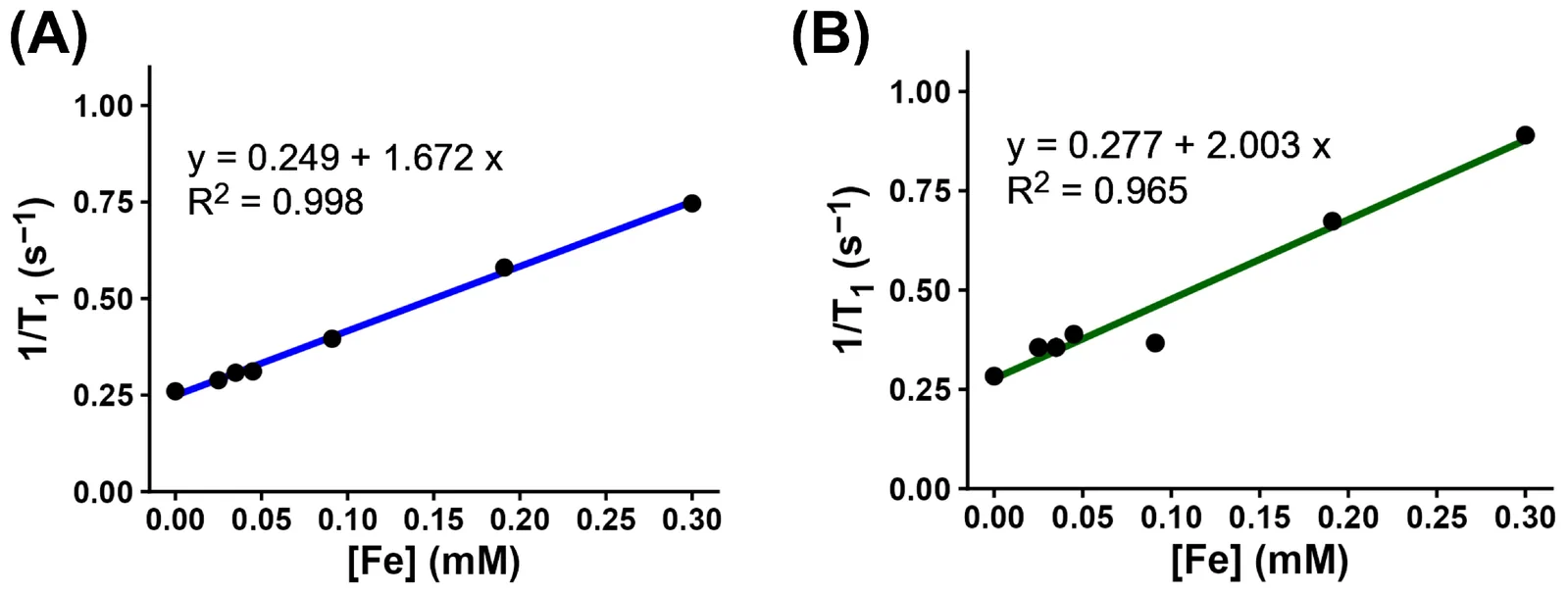
Transverse relaxation rates (1/*T*_1_, s^−1^) as a function of Fe concentration (mM) in (**A**) Mh@PECA and (**B**) (Mh@PECA)@Cs NPs, measured at 1.41 T. Black circles represent the experimental data, expressed as mean ± SD (*n* = 3), and solid lines represent the corresponding linear fits.

The longitudinal (*r*_1_) and transverse (*r*_2_) relaxivity were determined from the slopes of three independent linear regressions of 1/*T*_1_ and 1/*T*_2_ as a function of Fe concentration, respectively, each corresponding to an experimental replicate. The reported relaxivity values represent the means ± SD of these independent determinations (Table 6), whereas Figure 7 and Figure 8 show representative averaged relaxation profiles for visualization purposes.

The Mh@PECA NPs exhibited *r*_2_ = 97.66 ± 1.34 mM^−1^ s^−1^, while chitosan functionalization produced a marked increase to 236.02 ± 3.62 mM^−1^ s^−1^ for the (Mh@PECA)@Cs NPs (Table 5). Since these measurements were performed at 1.41 T, a magnetic field strength close to the clinical field of 1.5 T, direct comparison with clinically approved iron oxide-based MRI contrast agents is appropriate. The *r*_2_ value obtained for Mh@PECA is comparable to that reported for Ferumoxytol^®^ (≈80–100 mM^−1^ s^−1^), whereas that for the (Mh@PECA)@Cs NPs exceeds the relaxivity reported for Resovist^®^ (≈150–190 mM^−1^ s^−1^), Endorem^®^/Feridex^®^ (≈90–120 mM^−1^ s^−1^) and Sinerem^®^/Combidex^®^ (≈60–70 mM^−1^ s^−1^), indicating a substantial improvement in *T*_2_ contrast efficiency.

The *r*_1_ were considerably lower, reaching 1.66 ± 0.001 and 1.99 ± 0.015 mM^−1^ s^−1^ for the Mh@PECA and (Mh@PECA)@Cs NPs, respectively (Table 6). Linear regression analysis showed excellent reproducibility, with *R*^2^ values above 0.99 and narrow 95% confidence intervals for all independent measurements. Consequently, *r*_2_/*r*_1_ ratios of 58.8 and 118.6 were obtained for the Mh@PECA and (Mh@PECA)@Cs NPs, respectively, confirming the suitability of both formulations as efficient *T*_2_-weighted MRI contrast agents. Notably, chitosan functionalization increased the *r*_2_/*r*_1_ ratio more than two-fold, demonstrating that the outer polysaccharide layer substantially enhances *T*_2_ contrast performance.

Importantly, the *r*_2_ value for the (Mh@PECA)@Cs NPs is comparable to those reported for highly efficient iron oxide nanostructures [15,63,64,65], including clustered SPIONs, nanoflowers, or multicore NPs which typically exhibit *r*_2_ values in the range of 200 and 300 mM^−1^·s^−1^. One possible explanation for the enhanced *r*_2_ relaxivity observed in the (Mh@PECA)@Cs NPs is related to the structural characteristics of the chitosan coating. Previous studies have demonstrated that the compactness and effective thickness of polymer coatings strongly influence relaxivity, since the coating acts as a physical barrier that increases the minimum approach distance of water protons to the magnetic core [66,67]. Accordingly, the relatively open structure of chitosan may facilitate water diffusion in the vicinity of the magnetic core, thereby promoting proton dephasing and contributing to the high *r*_2_ values observed in this study. However, this hypothesis is based on the physicochemical properties of the coating and literature reports, and additional experimental studies would be required to elucidate the exact mechanism responsible for the observed relaxometric enhancement.

#### 3.2.6. MRI Phantom Imaging

**Figure 9:**
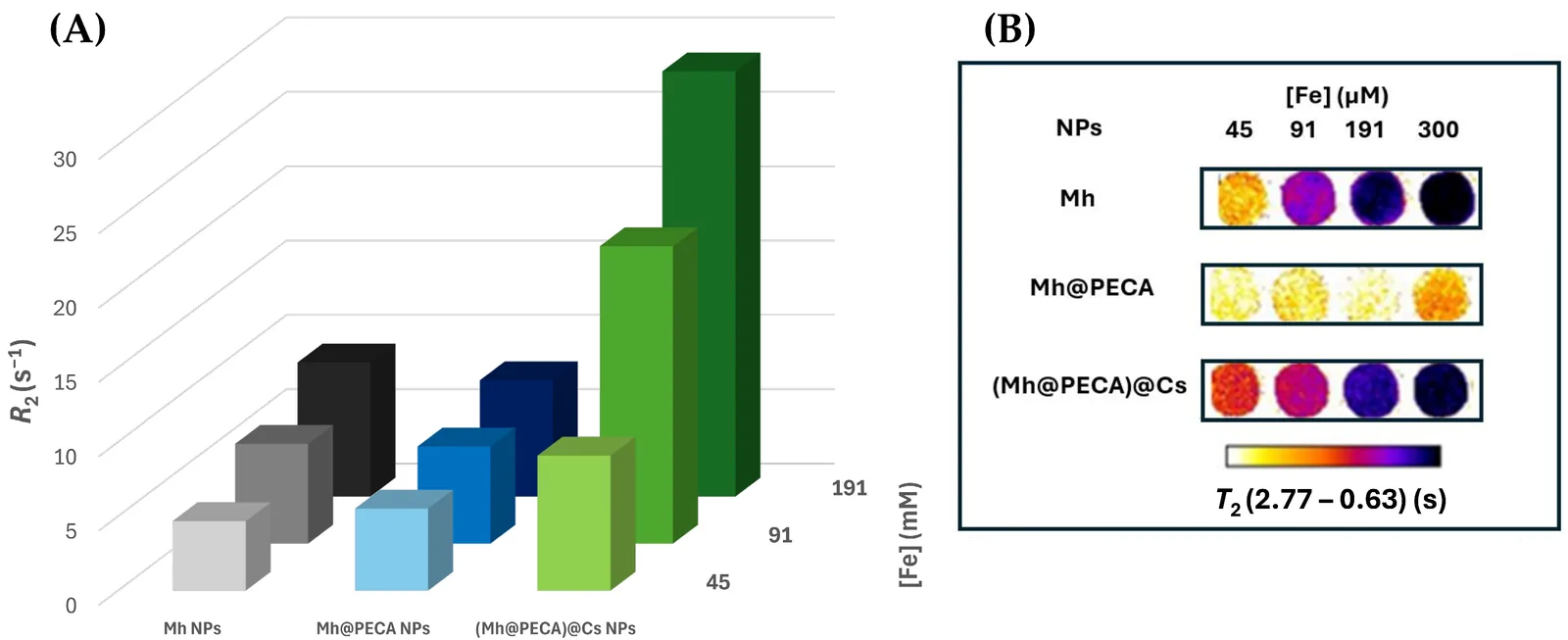
(**A**) Histogram *R*_2_ values obtained from *T*_2_-map MR phantom images (3.0 T) of aqueous NP dispersion ([Fe] = 45 to 191 μM) at pH 5.5 and 25 °C. Gray, blue, and green histograms correspond to Mh, Mh@PECA, and (Mh@PECA)@Cs NPs, respectively; within each color scale, darker shades indicate increasing Fe concentrations, as detailed in the legend. (**B**) *T*_2_-map MR phantom images (3.0 T) of aqueous NP dispersion ([Fe] = 45 to 300 μM).

MRI phantom experiments performed at 3 T were consistent with the relaxometric characterization obtained at 1.41 T (Table 6). At equivalent iron concentrations, (Mh@PECA)@Cs NPs generated markedly higher transverse relaxation rates (*R*_2_) values than both bare magnetic cores and Mh@PECA NPs (Figure 9A), resulting in progressively stronger negative *T*_2_-weighted contrast in the corresponding phantom images (Figure 9B).

This enhancement became more pronounced with increasing iron concentration, demonstrating that chitosan functionalization significantly improves the *T*_2_-weighted contrast efficiency under clinically relevant imaging conditions.

To enable a direct comparison of the *T*_2_-weighted contrast performance, phantom MRI images were acquired using identical Fe concentrations (45, 91, 191, and 300 μM) for Mh, Mh@PECA, and (Mh@PECA)@Cs NPs under the same imaging conditions (3 T). This concentration-dependent phantom study provides a direct comparison of the contrast efficiency of the three formulations while excluding differences arising from iron concentration or imaging conditions.

The concentration-dependent increase in *R*_2_ observed for all formulations is in good agreement with the relaxivity measurements (Table 6). Notably, (Mh@PECA)@Cs NPs consistently exhibited the highest *R*_2_ values throughout the investigated concentration range, corroborating the superior *T*_2_-weighted imaging performance predicted from their elevated *r*_2_ relaxivity. This enhanced MRI contrast is consistent with the relaxometric characterization and supports the beneficial effect of Cs functionalization on the imaging performance of the proposed nanoplatform. Although the structural characteristics of the Cs coating may contribute to the observed enhancement, additional studies will be required to fully elucidate the underlying mechanism.

### 3.3. Evaluation of Hemocompatibility

The hemocompatibility of the Mh@PECA-based nanoplatforms was systematically evaluated through hemolysis, platelet activation, complement activation, and stability assays (Table 7). Both Mh@PECA and (Mh@PECA)@CsNPs exhibited low hemolytic activity over time, with values remaining in the range of 2.4–2.7%, corresponding to slightly hemolytic behavior according to ISO 10993-4, but still within acceptable limits for nanomaterial systems. Platelet activation, assessed via P-selectin release, showed comparable values for both formulations (113 ± 5 and 113 ± 6 ng/mL) relative to the PBS control (99 ± 8 ng/mL), indicating only a modest increase (~1.1–1.2-fold) and thus low thrombogenic potential. Similarly, complement activation, evaluated through C3a desArg levels, revealed minimal differences between NP-treated samples (302–303 ng/mL) and control (294 ± 8 ng/mL), suggesting limited activation of the complement cascade. The circulation half-life (t_1_/_2_ max) was slightly increased for both NPs (13.9–14.2 min) compared with PBS (11.2 ± 1.9 min), indicating good colloidal stability without substantial alteration in clearance behavior under simplified conditions. Notably, Cs functionalization did not significantly affect any of the evaluated hemocompatibility parameters. Overall, these findings indicate no evidence of significant adverse interactions with the evaluated blood components under the experimental conditions tested. These encouraging preliminary findings are consistent with the continued development of this nanoplatform for MRI-guided magnetic hyperthermia applications [45,68,69].

The hemocompatibility assessment presented herein should be considered a preliminary ex vivo evaluation intended to identify potential acute blood compatibility issues during the early development of the proposed nanoplatform. Accordingly, these findings should not be interpreted as a comprehensive blood compatibility assessment. Future preclinical studies should include larger donor cohorts together with additional blood compatibility assays and appropriate positive controls to further establish the hemocompatibility profile of the proposed nanosystem. Similar stepwise validation strategies have been adopted in previous proof-of-concept studies involving magnetic and metallic NPs [70,71,72].

### 3.4. MRI Proof of Concept: Cellular MRI Visualization of HeLa Cells Following NP Uptake

**Figure 10:**
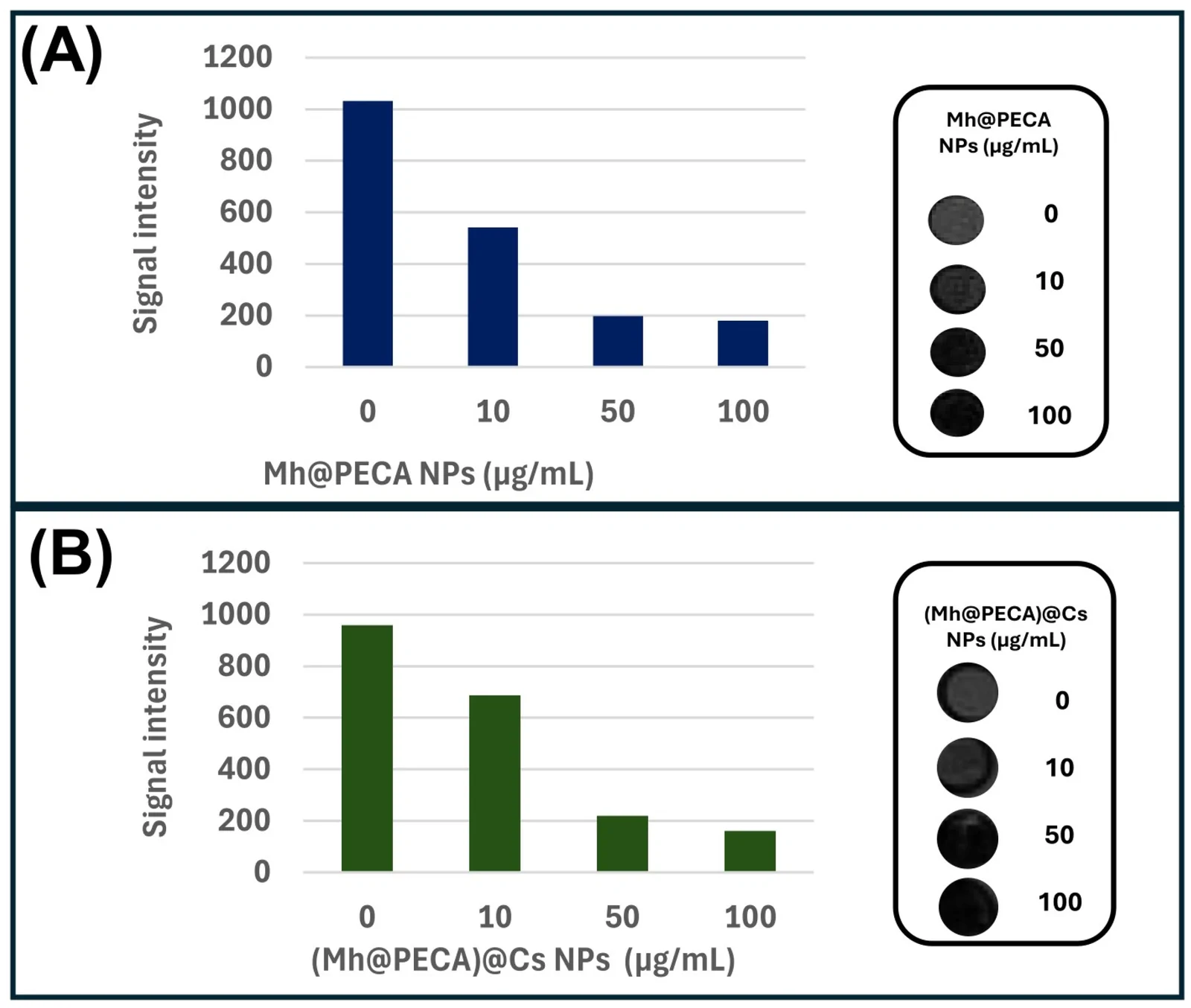
MR phantoms of HeLa cells incubated with different NP formulations and corresponding quantitative analysis. (**A**) Signal intensity (arbitrary units) as a function of NP concentration (0–100 µg/mL) for Mh@PECA NPs after incubation with HeLa cells, including untreated cells as control. (**B**) Signal intensity (arbitrary units) versus NP concentration (0–100 µg/mL) for (Mh@PECA)@Cs NPs after incubation with HeLa cells, including control cells. In all panels, representative MR phantom images are shown on the right, while the corresponding quantitative signal intensity values (arbitrary units) as a function of NP concentration are presented on the left.

NP internalization was indirectly assessed by MRI in HeLa cell pellets after incubation at different concentrations (10, 50, and 100 µg/mL) (Figure 10). Both Mh@PECA NPs and (Mh@PECA)@Cs NPs generated detectable contrast in cellular phantoms, due to efficient cellular internalization, compared with the control (cells without NPs). In both cases, contrast increased with iron concentration, as reflected by a progressive decrease in *T*_2_ signal intensity. This effect became more pronounced with the increase in NP concentration, and thus iron content, in the assay. Notably, a significant reduction in signal intensity compared with water (≈1000) was already observed even at the lowest tested concentration (10 μg/mL) for all NPs. The lowest signal intensity, corresponding to the highest contrast, was consistently observed in wells containing 100 μg/mL (Figure 10A,B).

**Figure 11:**
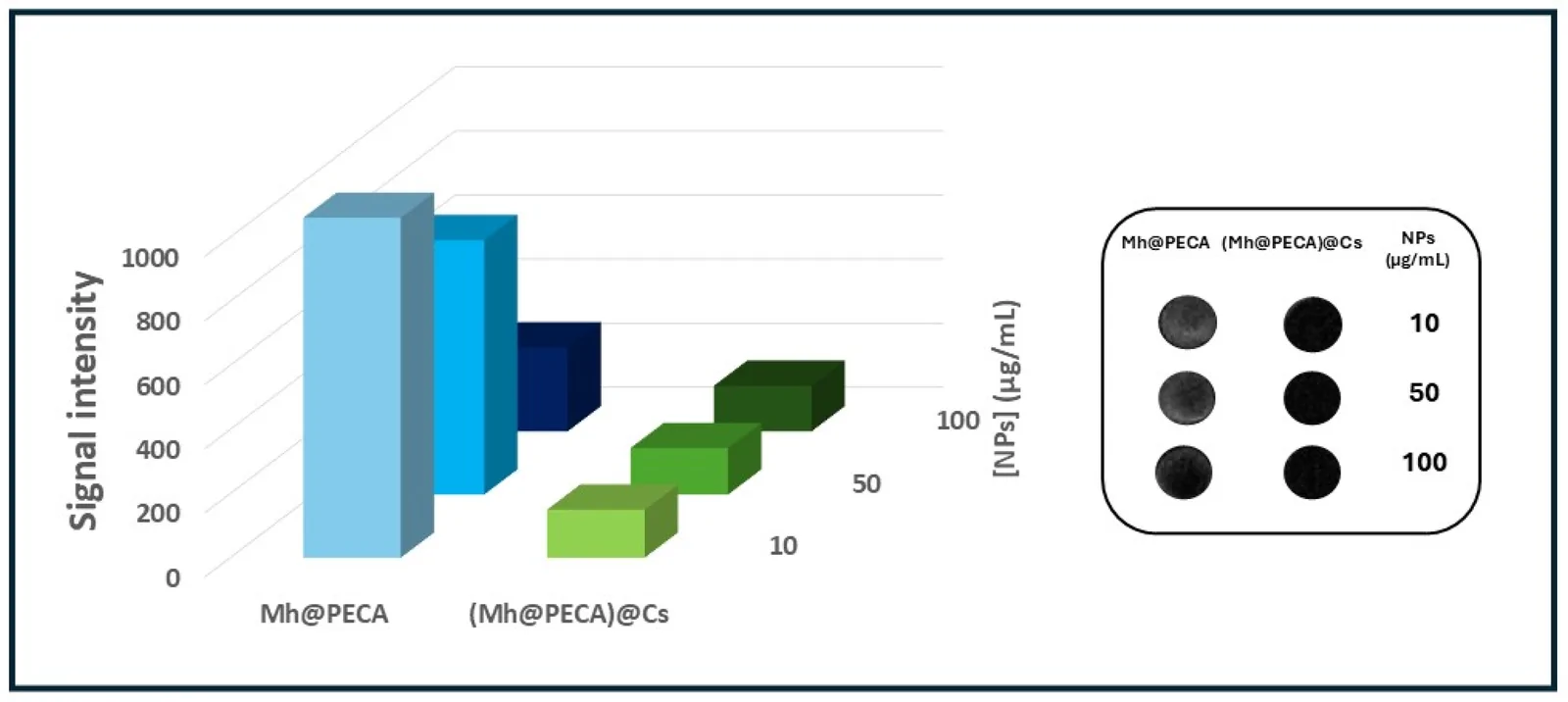
Representative MR phantom images (**right**) of HeLa cells incubated for 48 h with Mh@PECA and Mh@PECA@Cs NPs at iron concentrations of 10, 50, and 100 µg Fe/mL, together with the corresponding quantitative analysis of MR signal intensity (**left**). Signal intensity values are expressed in arbitrary units (a.u.). In the histograms shown on the left, blue and green shades represent Mh@PECA and (Mh@PECA)@Cs NPs, respectively. Within each color scale, lighter shades correspond to the lowest iron concentration (10 µg Fe/mL), whereas darker shades correspond to the highest concentration (100 µg Fe/mL), as indicated in the legend.

In a third experiment (Figure 11), Mh@PECA NPs and (Mh@PECA)@Cs NPs were compared within the same MRI phantom. This comparison appears to be consistent with the relaxometry data, as the contrast was significantly higher in HeLa cells incubated with (Mh@PECA)@Cs NPs than in those incubated with Mh@PECA NPs.

### 3.5. Quantification of NP Internalization in HeLa Cells by Fe Content Determination

The internalization capacity of the (Mh@PECA)@Cs NPs was evaluated by quantifying intracellular Fe concentration after incubation of 10^6^ HeLa cells with 100 μg/mL NPs.

After incubation for 24 h, several washing steps were performed to remove extracellular NPs, and Fe content was quantified by intracellular iron quantification (ICP) in HeLa cells incubated with NPs and in untreated cells (control).

An intracellular Fe content of 7.646 ± 0.395 pg per cell was quantified. After subtracting the endogenous Fe content measured in control cells and considering the amount of Fe added in the form of NPs, Fe internalization efficiency in HeLa cells was found to be 53.62 ± 3.14%.

Efficient internalization of iron oxide NPs in HeLa cells has been widely reported, mainly through macropinocitosis or endocytosis-mediated mechanisms [73]. The intracellular Fe amount reported in this study was ~7.65 pg Fe per cell, which was significantly higher than the control, ~0.89 pg Fe/cell, falling within the range reported in the literature for cellular uptake of iron-based NPs [74].

### 3.6. Evaluation of Hyperthermia

#### 3.6.1. Heating Performance Under AMF

**Figure 12:**
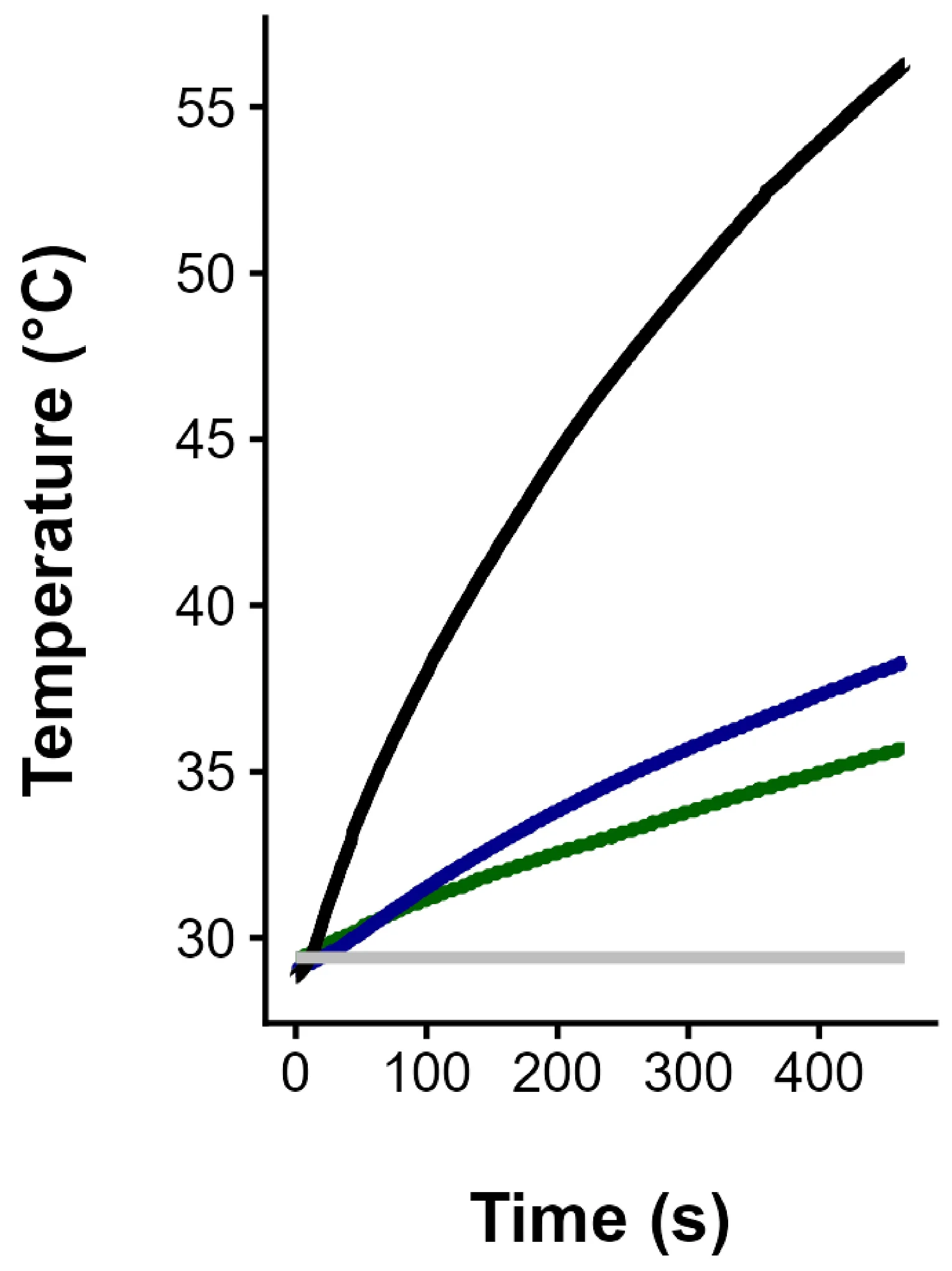
Heating profiles of Mh NPs (black line), Mh@PECA NPs (blue line) at an iron concentration of 2.3 mg Fe mL^−1^, and (Mh@PECA)@Cs NPs (green line) at an iron concentration of 0.235 mg Fe mL^−1^ under an AMF (20 mT, 869 kHz). The gray line corresponds to the heating profile of water (negative control).

Bare Mh NPs reached the hyperthermia threshold temperature (42 °C) within 2.87 min at a Fe concentration of 2.3 mg/mL. Under the same iron concentration, Mh@PECA NPs required a significantly longer time (14.17 min) to reach 42 °C. In contrast, (Mh@PECA)@Cs NPs achieved the hyperthermia temperature after 22.34 min despite containing a tenfold lower iron concentration (0.235 mg/mL) (Figure 12). From a biomedical perspective, achieving therapeutic temperatures with reduced Fe content is highly advantageous, as it may minimize potential toxicity associated with high metal doses while maintaining effective heat generation.

In agreement with these observations, SAR values (Table 8) revealed notable differences among the formulations. The Mh NPs exhibited an SAR of 147.2 ± 0.4 W/g Fe, whereas the Mh@PECA NPs showed a significantly lower value (49.1 ± 0.1 W/g Fe). Conversely, the (Mh@PECA)@Cs NPs displayed the highest SAR (309.2 ± 0.8 W/g Fe), despite their lower iron concentration. The corresponding ILP values were 0.67, 0.22, and 1.34 nH·m^2^·kg^−1^ for the Mh, Mh@PECA, and (Mh@PECA)@Cs NPs, respectively, confirming that the superior heating performance of the chitosan-coated formulation is maintained after normalization for the applied magnetic field amplitude and frequency. These differences in heating efficiency can be explained by magnetic relaxation processes, namely, Néel and Brownian relaxation, through which magnetic energy is dissipated as heat [12,75]. The relative contribution of these mechanisms governs the heating behavior and depends strongly on particle size, hydrodynamic volume, and the physicochemical properties of the local environment, particularly viscosity and confinement [15,76]. Accordingly, the reduced SAR observed for Mh@PECA NPs may be attributed to increased interparticle interactions and restricted mobility induced by the polymeric coating, which can hinder effective magnetic relaxation [77].

Furthermore, the enhanced heating performance observed for (Mh@PECA)@Cs NPs may be associated with modifications in the local microenvironment induced by the chitosan coating. Specifically, an increase in viscosity may hinder Brownian rotation and promote Néel relaxation as the dominant heat dissipation mechanism, thereby explaining the higher SAR values obtained for this formulation [55,57,75,78].

#### 3.6.2. Induced Effects in HeLa Cells

**Figure 13:**
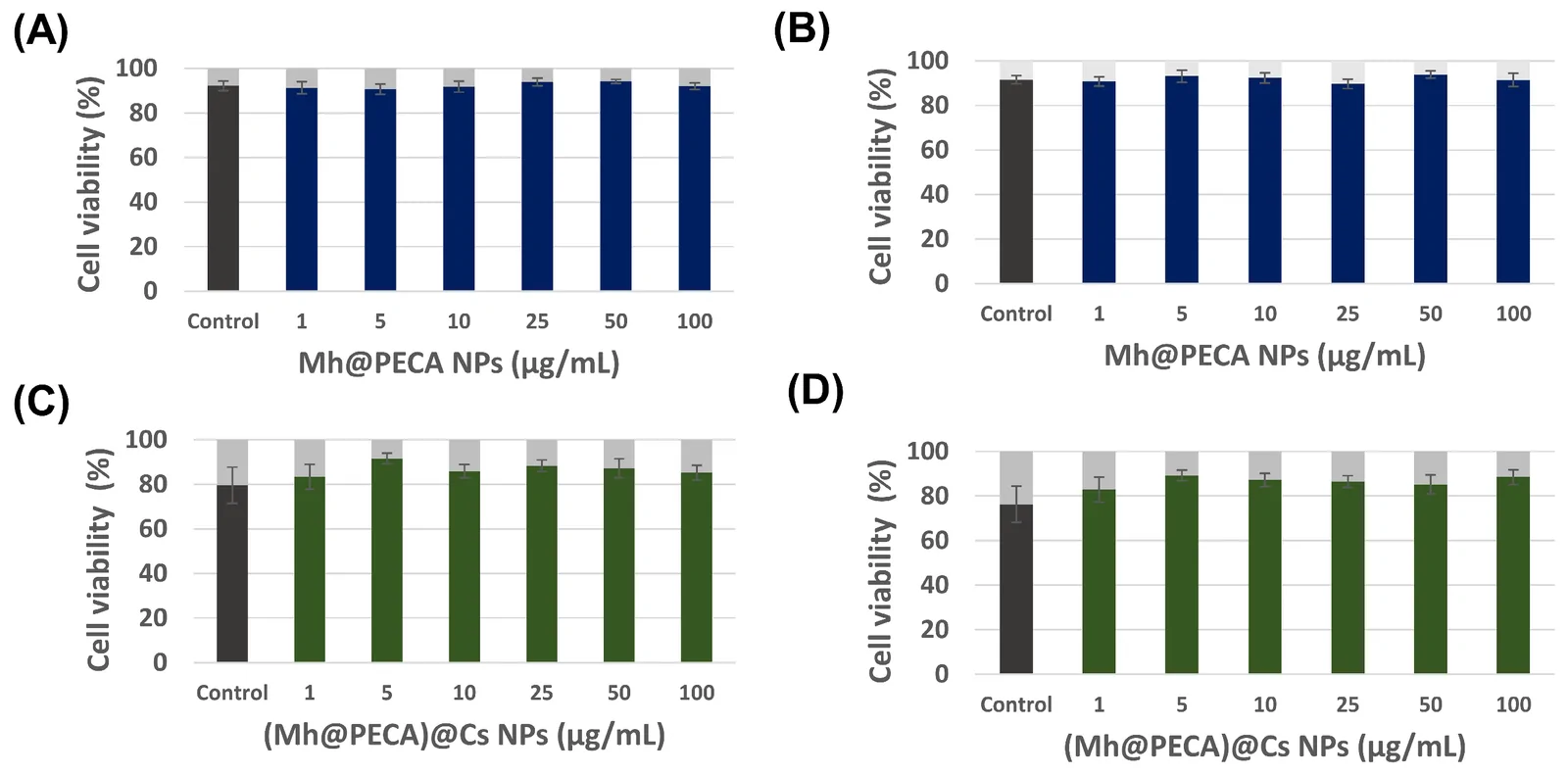
Cell viability of HeLa cells after 24 h (**A**,**C**) and 48 h (**B**,**D**) of exposure to Mh@PECA NPs (**A**,**B**) and (Mh@PECA)@Cs NPs (**C**,**D**) at concentrations up to 100 µg/mL. Blue bars represent cells exposed to Mh@PECA NPs (**A**,**B**), green bars represent cells exposed to (Mh@PECA)@Cs NPs (**C**,**D**), and gray bars represent the control group (HeLa cells without NPs).

Cytocompatibility of the Mh@PECA NPs was evaluated in HeLa cells after incubation during 24 h (Figure 13A) and 48 h (Figure 13B). Cell viability remained close to control values (~90%) under the tested conditions. Similar outcomes were obtained for the (Mh@PECA)@Cs NPs after 24 h (Figure 13C) and 48 h (Figure 13D), indicating that Cs functionalization did not induce additional baseline cytotoxicity.

**Figure 14:**
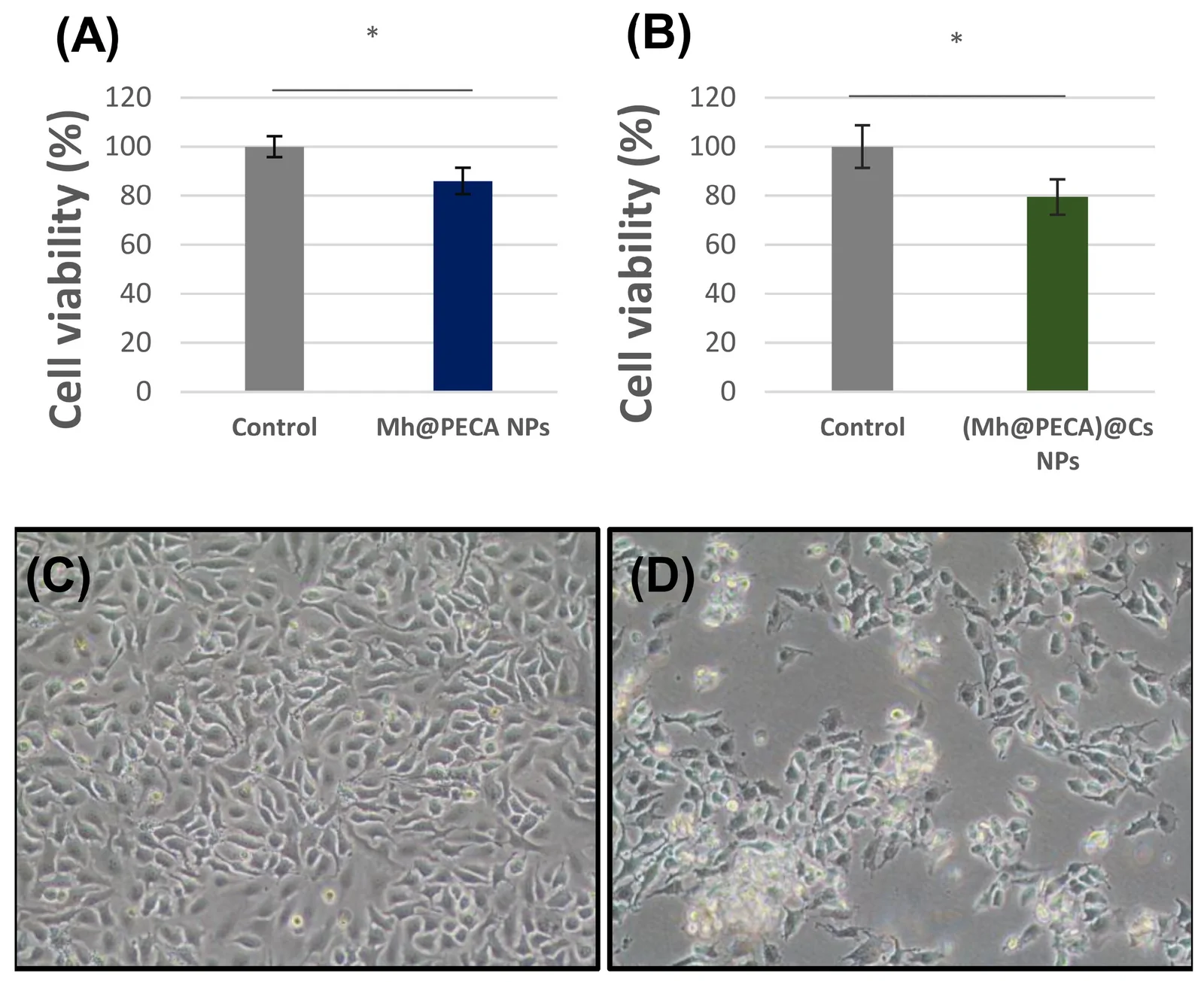
Cell viability (%) of HeLa cells exposed to an AMF in the absence of (control) and with Mh@PECA NPs (**A**) and (Mh@PECA)@Cs NPs (**B**) concentrations of up to 100 µg/mL. In panels (**A**) and (**B**), gray bars represent the control group (HeLa cells exposed to the AMF without NPs), whereas blue and green bars represent cells treated with Mh@PECA and (Mh@PECA)@Cs NPs, respectively. (**C**) Optical microscopy image (10×) of control HeLa cells exposed to an AMF in the absence of NPs. (**D**) Optical microscopy image (10×) of HeLa cells treated with (Mh@PECA)@Cs NPs after AMF exposure, showing reduced cell density and morphological alterations associated with hyperthermia-induced effects. * Statistically significant differences were observed (*p* < 0.05).

Upon application of the AMF (Figure 14), a statistically significant reduction (*p* < 0.05) in cell viability was observed for both NP systems compared with control HeLa cells not exposed to NPs (Figure 14A,B). This decrease confirms the induction of a hyperthermic effect under the applied conditions. Cell viability was 85.99 ± 5.43% for the Mh@PECA NPs and 79.48 ± 7.22% for the (Mh@PECA)@Cs NPs under AMF exposure.

These findings are supported by the optical microscopy images (bright-field) shown in Figure 14C,D, where pronounced morphological alterations can be observed after AMF exposure. Cells treated with (Mh@PECA)@Cs NPs under the AMF exhibited a reduced cell density, loss of the typical elongated morphology, and increased presence of rounded and detached cells, all of which are indicative of cell damage and loss of viability (Figure 14D). In contrast, cells not exposed to NPs retained their normal morphology (Figure 14C).

Overall, these results indicate that both NP systems induce a statistically significant hyperthermic effect on HeLa cells, as reflected by the reduction in cell viability relative to control conditions. This effect can be attributed to AMF-induced hyperthermia, as evidenced by the difference in cell viability observed under AMF exposure in the absence of NPs (Figure 14).

The present biological evaluation represents an initial assessment of the theranostic potential of the developed nanoplatform. HeLa cells were selected as a representative tumor model because they are widely employed in studies of magnetic hyperthermia and MNP-mediated therapies, facilitating comparison with previously reported systems [26,39,40,41,42]. Although extending the biological evaluation to non-malignant cell models would provide valuable information regarding the safety profile of the proposed nanosystem, the cellular response to MNPs is known to depend on factors such as tissue origin, phenotype, metabolic activity, and NP uptake [40]. Consequently, no single non-malignant cell line can comprehensively represent the behavior of healthy tissues. Future studies will therefore expand the biological evaluation to additional tumor models with different thermosensitivity, as well as to appropriate non-malignant cell lines and complementary biological assays, including apoptosis/necrosis analyses, cellular uptake and intracellular localization studies, to further investigate the therapeutic selectivity, mechanism of action, and safety profile of the proposed nanoplatform.

## 4. Conclusions

In this work, a previously unexplored (Mh@PECA)@Cs hybrid nanoplatform was successfully developed and optimized through systematic variation in Fe core, ECA monomer, and Cs mass ratios, enabling control over key properties such as particle size and PdI. Comprehensive characterization supports the formation of a (core/shell)/shell nanostructure that preserved the superparamagnetic behavior of the magnetic core. This magnetic responsiveness enabled efficient heat generation under an AMF, as well as measurable *T*_2_-weighted MRI contrast enhancement in both aqueous media and HeLa cell-based models. Notably, Cs functionalization led to a significant enhancement in MRI contrast performance, supporting the contribution of the outer coating to the transverse relaxometric properties of the NPs. Preliminary biological evaluation revealed no evidence of significant adverse hemocompatibility under the experimental conditions evaluated, together with efficient cellular internalization, and a statistically significant reduction in HeLa cell viability following AMF exposure. Collectively, these findings support the feasibility of combining *T*_2_-weighted MRI contrast enhancement and magnetic hyperthermia within a single biodegradable nanoplatform.

Overall, these results demonstrate the preliminary in vitro potential of the (Mh@PECA)@Cs nanocomposite as a multifunctional magnetic platform for potential MRI-guided magnetic hyperthermia applications. The ability to combine efficient *T*_2_-weighted MRI contrast enhancement with AMF-induced cytotoxicity highlights its promise as a candidate theranostic platform. However, these findings should be regarded as proof-of-concept evidence and do not yet establish its suitability as a theranostic platform for clinical use. Moreover, the response to magnetic hyperthermia depends strongly on tumor type and cellular thermosensitivity [26,79]. Therefore, the moderate hyperthermic effect observed in HeLa cells should not be considered representative of all cancer models, and future studies will evaluate the therapeutic efficacy of this nanoplatform in additional tumor cell lines exhibiting different thermal sensitivity.

In vivo, magnetic hyperthermia may be achieved by exposing the tumor region containing the accumulated MNPs to an externally applied AMF, thereby promoting localized heat generation. This treatment strategy has already reached clinical application for selected cancers using dedicated AMF systems [26,80]. Nevertheless, the clinical translation of the present nanoplatform will require comprehensive in vivo evaluation of its biodistribution, pharmacokinetics, clearance, tumor accumulation, therapeutic efficacy, and short- and long-term safety. Complementary in vitro studies, including apoptosis and necrosis analyses, additional viability assays, and detailed intracellular localization studies, will also be required to characterize its biological effects more fully.

## Figures and Tables

**Table 1:** Influence of Mh:ECA mass ratio on particle size and PdI (*n* = 3).

Mh:ECA Ratio	Size (nm) ± SD	PdI ± SD
2:4	228.81 ± 9.97	0.334 ± 0.058
3:4	356.81 ± 6.22	0.275 ± 0.018
4:4	450.91 ± 21.26	0.341 ± 0.004
4:3	655.21 ± 21.64	0.363 ± 0.005
4:2	391.21 ± 11.46	0.375 ± 0.004

**Table 2:** Effect of (Mh@PECA):Cs mass ratio on particle size and PdI (*n* = 3).

(Mh@PECA):Cs Ratio	Size (nm) ± SD	PdI ± SD
2:4	341.65 ± 84.27	0.318 ± 0.025
3:4	376.45 ± 111.91	0.212 ± 0.067
4:4	543.50 ± 154.31	0.070 ± 0.008
4:3	412.90 ± 122.51	0.160 ± 0.106
4:2	304.05 ± 107.41	0.215 ± 0.028

**Table 3:** Hydrodynamic diameter (nm), PdI, *ζ* (mV), and pH values of Mh@PECA and (Mh@PECA)@Cs NPs as a function of storage time (days) at 4.0 ± 0.5 °C and 25.0 ± 0.5 °C. Data are presented as means ± standard deviation (SD) (*n* = 3).

Stability Assay at 25.0 ± 0.5 °C								
Time (Days)	Mh@PECA NPs				(Mh@PECA)@Cs NPs			
	Size (nm)	PdI	*ζ* (mV)	pH	Size (nm)	PdI	*ζ* (mV)	pH
0	234.87 ± 1.72	0.321 ± 0.024	−13.57 ± 0.55	6.13	348.40 ± 47.80	0.420 ± 0.040	9.33 ± 0.26	4.67
2	229.07 ± 8.98	0.356 ± 0.056	−9.08 ± 0.97	6.14	381.27 ± 10.17	0.289 ± 0.056	11.47 ± 1.00	4.90
4	213.30 ± 12.98	0.365 ± 0.061	−3.72 ± 0.66	6.14	464.23 ± 9.42	0.345 ± 0.030	12.03 ± 0.46	4.28
7	222.53 ± 3.44	0.397 ± 0.017	−13.2 ± 0.82	6.11	292.97 ± 41.20	0.396 ± 0.014	13.90 ± 0.30	4.30
9	282.00 ± 24.47	0.437 ± 0.086	−6.67 ± 1.17	5.76	365.63 ± 13.48	0.373 ± 0.029	11.27 ± 0.91	4.29
14	361.43 ± 9.69	0.493 ± 0.091	−8.19 ± 0.81	5.42	394.33 ± 18.08	0.454 ± 0.044	9.73 ± 2.39	4.36
16	397.40 ± 51.47	0.564 ± 0.018	−6.26 ± 1.18	5.37	403.73 ± 24.92	0.470 ± 0.110	11.67 ± 0.83	4.49
18	295.27 ± 23.51	0.431 ± 0.037	−11.00 ± 0.53	5.41	376.10 ± 14.65	0.467 ± 0.015	12.47 ± 0.60	4.55
21	288.37 ± 41.15	0.489 ± 0.080	−6.37 ± 1.38	5.55	404.30 ± 16.95	0.522 ± 0.012	11.71 ± 2.04	4.71
25	288.37 ± 41.15	0.453 ± 0.054	−6.11 ± 0.02	5.70	396.23 ± 10.46	0.489 ± 0.012	9.72 ± 0.46	5.38
**Stability Assay at 4.0 ± 0.5 °C**								
**Time (Days)**	**Mh@PECA NPs**				**(Mh@PECA)@Cs NPs**			
	**Size (nm)**	**PdI**	***ζ* (mV)**	**pH**	**Size (nm)**	**PdI**	***ζ* (mV)**	**pH**
0	234.87 ± 1.72	0.321 ± 0.024	−13.57 ± 0.55	6.13	348.40 ± 47.80	0.425 ± 0.261	9.33 ± 0.26	4.66
2	215.03 ± 4.39	0.365 ± 0.013	−9.47 ± 0.18	6.08	346.13 ± 3.88	0.232 ± 0.021	12.26 ± 0.75	4.44
4	230.50 ± 7.10	0.363 ± 0.016	−3.19 ± 0.12	6.31	432.42 ± 4.04	0.300 ± 0.040	11.86 ± 0.40	4.37
7	226.80 ± 10.42	0.349 ± 0.440	−8.74 ± 5.22	6.17	408.53 ± 22.23	0.364 ± 0.027	15.17 ± 0.55	4.35
9	251.73 ± 8.34	0.438 ± 0.044	−4.50 ± 1.24	6.08	420.03 ± 9.00	0.317 ± 0.061	10.40 ± 0.43	4.32
14	220.00 ± 3.63	0.376 ± 0.020	−7.98 ± 0.58	6.02	393.57 ± 8.94	0.305 ± 0.033	10.83 ± 0.38	4.35
16	239.4 ± 8.56	0.415 ± 0.033	−6.21 ± 0.32	5.96	405.50 ± 12.99	0.307 ± 0.058	14.20 ± 1.57	4.34
18	261.03 ± 14.26	0.430 ± 0.075	−5.75 ± 0.61	6.07	370.43 ± 27.98	0.373 ± 0.047	10.40 ± 0.97	4.43
21	251.97 ± 5.29	0.433 ± 0.019	−13.16 ± 0.75	5.93	406.93 ± 16.10	0.346 ± 0.075	12.67 ± 1.10	4.42
25	248.44 ± 12.33	0.437 ± 0.164	−15.41 ± 0.76	6.11	394.29 ± 20.68	0.342 ± 0.033	12.42 ± 1.91	4.40

**Table 4:** Hydrodynamic diameter (nm), PdI, *ζ* (mV) of (Mh@PECA)@Cs NPs in PBS (pH 7.0) as a function of storage time (hours) at 25.0 ± 0.5 °C and 72.0 ± 0.5 °C. Data are presented as means ± standard deviation (SD) (*n* = 3).

Time	Temperature (°C)	Size	PdI	*ζ* (mV)
24 h	25.0	339.47 ± 5.12	0.317 ± 0.003	17.40 ± 0.98
	37.0	359.20 ± 6.03	0.390 ± 0.044	13.77 ± 0.25
48 h	25.0	304.33 ± 4.01	0.171 ± 0.067	15.33 ± 1.37
	37.0	291.97 ± 8.12	0.288 ± 0.051	12.67 ± 0.52
120 h	25.0	277.40 ± 5.40	0.291 ± 0.030	13.40 ± 0.22
	37.0	300.80 ± 5.68	0.259 ± 0.024	11.07 ± 0.45

**Table 5:** Magnetic parameters characteristic of the NPs.

Nanomaterial	*M*_s_ (Am^2^/kg)	*χ*_0_ (10^−3^) (m^3^/kg)
Mh	15.86 ± 0.77	0.218 ± 0.034
Mh@PECA	10.05 ± 0.47	0.142 ± 0.019
(Mh@PECA)@Cs	8.15 ± 0.38	0.115 ± 0.016

**Table 6:** Longitudinal ($r_{1}$) and transverse ($r_{2}$) relaxivity of Mh@PECA and (Mh@PECA)@Cs NPs calculated from three independent linear regressions of $1/T_{1}$ and $1/T_{2}$ as a function of Fe concentration. Individual slope values, means ± SD, 95% confidence intervals (95% CIs), and coefficients of determination ($R^{2}$) are reported.

Nanomaterial	Type	Replicate 1	Replicate 2	Replicate 3	Mean ± SD	95% CI	*R* ^2^
Mh@PECA	*r*_1_	1.668	1.669	1.671	1.669 ± 0.001	1.665–1.673	0.9973–0.9971
Mh@PECA	*r*_2_	98.43	96.11	98.43	97.66 ± 1.340	94.32–100.99	0.9982–0.9989
(Mh@PECA)@Cs	*r*_1_	2.008	1.976	1.992	1.992 ± 0.015	1.953–2.032	0.9978–0.9968
(Mh@PECA)@Cs	*r*_2_	233.77	234.09	240.19	236.02 ± 3.620	227.03–245.00	0.9962–0.9986

**Table 7:** Blood compatibility of the Mh@PECA NPs and (Mh@PECA)@Cs NPs: hemolysis (%), platelet activation (sP-selectin release, ng/mL), complement activation (C3a release: C3a desArg, ng/mL), and PRT (T_1/2_ max, min). Hemolysis values were normalized using PBS-treated erythrocytes as the negative control (0% hemolysis) and Triton X-100-treated erythrocytes as the positive control (100% hemolysis). Data are expressed as mean values ± SD (*n* = 3). PBS was used as the negative control in all assays. TRAP-6, zymosan, and kaolin were included as positive controls for platelet activation, complement activation, and coagulation, respectively.

**Sample**	**Hemolysis (%)**				**sP-Selectin Release (ng/mL)**	**C3a** **desArg (ng/mL)**	***T*_1/2 max_ (Min)**
	**Incubation Time**						
	2 h	4 h	8 h	24 h			
Mh@PECA NPs	2.5 ± 0.6	2.4 ± 0.5	2.6 ± 0.6	2.7 ± 0.3	113 ± 5	303 ± 6	13.9 ± 1.6
(Mh@PECA)@Cs NPs	2.6 ± 0.7	2.7 ± 0.6	2.4 ± 0.6	2.6 ± 0.9	113 ± 6	302 ± 12	14.2 ± 1.2
Negative control Positive control					99 ± 8	294 ± 8	11.2 ± 1.9
					285 ± 35	780 ± 90	5.3 ± 0.4

**Table 8:** Comparison of SAR and ILP values ± SD (W/g Fe) for Mh, Mh@PECA, and (Mh@PECA)@Cs NPs.

Nanomaterial	SAR (W/g Fe)	ILP (nHm^2^)/kg
Mh	147.20 ± 0.40	0.67 ± 0.002
Mh@PECA	49.10 ± 0.10	0.223 ± 0.001
(Mh@PECA)@Cs	309.20 ± 0.80	1.406 ± 0.004

## Data Availability

The data presented in this study are available upon request from the corresponding author.

## Abbreviations

The following abbreviations are used in this manuscript:

AMF	Alternating magnetic field
*AT*	Acquisition time
BSA	Bovine serum albumin
Cs	Chitosan
DMEM	Dulbecco’s modified eagle’s medium
ECA	Ethyl cyanoacrylate
EDX	Energy-Dispersive X-ray Spectroscopy
EPR	Enhanced permeability and retention
FBS	Fetal bovine serum
*FOV*	Field of View
FTIR	Fourier transform infrared spectroscopy
HAADF-STEM	High-Angle Annular Dark-Field Scanning Transmission Electron Microscopy
HBSS	Hank’s Balanced Salt Solution
ICP	Intracellular iron quantification
ICP-HRMS	Inductively coupled plasma high-resolution mass spectrometry
ILP	intrinsic loss power
Mh	Maghemite
MNPs	Magnetic nanoparticles
MRI	Magnetic resonance imaging
*M*_s_	Saturation magnetization
*NA*	Number of averages
*NE*	Number of echoes
NPs	Nanoparticles
*Ns*	Number of slices
PACA	Poly(alkyl cyanoacrylate)
PBS	Phosphate-buffered saline
PdI	Polydispersity index
PECA	Poly(ethyl cyanoacrylate)
PEG	Polyethylene glycol
PRT	Plasma recalcification time
*r*_1_	Longitudinal relaxivity
*r*_2_	Transverse relaxivity
*R*_2_	Transverse relaxation rates
ROIs	Regions of interest
SAR	Specific absorption rates
SPIONs	Super paramagnetic iron oxide nanoparticles
*S*_T_	Slice thickness
*TE*	Echo time
TEM	Transmission electron microscopy
*TR*	Repetition time
*Ζ*	Zeta potential
*χ*_0_	Initial magnetic susceptibility
